# DNA Barcoding of Neotropical Sand Flies (Diptera, Psychodidae, Phlebotominae): Species Identification and Discovery within Brazil

**DOI:** 10.1371/journal.pone.0140636

**Published:** 2015-10-27

**Authors:** Israel de Souza Pinto, Bruna Dias das Chagas, Andressa Alencastre Fuzari Rodrigues, Adelson Luiz Ferreira, Helder Ricas Rezende, Rafaela Vieira Bruno, Aloisio Falqueto, José Dilermando Andrade-Filho, Eunice Aparecida Bianchi Galati, Paloma Helena Fernandes Shimabukuro, Reginaldo Peçanha Brazil, Alexandre Afranio Peixoto

**Affiliations:** 1 Lab. de Biologia Molecular de Insetos, Instituto Oswaldo Cruz, FIOCRUZ, Brasil Ave. 4365, 21040360, Rio de Janeiro, RJ, Brazil; 2 Unidade de Medicina Tropical, UFES, Marechal Campos Ave. 1468, 29043900, Vitória, Espírito Santo, Brazil; 3 Lab. de Doenças Parasitárias, Instituto Oswaldo Cruz, FIOCRUZ, Brasil Ave. 4365, 21040360, Rio de Janeiro, RJ, Brazil; 4 Instituto Nacional de Ciência e Tecnologia em Entomologia Molecular – INCT-EM/CNPq, Rio de Janeiro, Brazil; 5 Grupo de Estudos em Leishmanioses, Centro de Pesquisas René Rachou, FIOCRUZ, Augusto de Lima Ave. 1715, 30190002, Belo Horizonte, MG, Brazil; 6 Departamento de Epidemiologia, Faculdade de Saúde Pública, USP, Dr. Arnaldo Cerqueira Cesar Ave. 715, 01246904, São Paulo, SP, Brazil; Field Museum of Natural History, UNITED STATES

## Abstract

DNA barcoding has been an effective tool for species identification in several animal groups. Here, we used DNA barcoding to discriminate between 47 morphologically distinct species of Brazilian sand flies. DNA barcodes correctly identified approximately 90% of the sampled taxa (42 morphologically distinct species) using clustering based on neighbor-joining distance, of which four species showed comparatively higher maximum values of divergence (range 4.23–19.04%), indicating cryptic diversity. The DNA barcodes also corroborated the resurrection of two species within the shannoni complex and provided an efficient tool to differentiate between morphologically indistinguishable females of closely related species. Taken together, our results validate the effectiveness of DNA barcoding for species identification and the discovery of cryptic diversity in sand flies from Brazil.

## Introduction

Sand flies (Diptera, Psychodidae, Phlebotominae) are small insects that are the main vectors of *Leishmania* Ross parasites, the etiologic agents of the leishmaniases. These insects are also vectors of other pathogens, including *Bartonella* Strong *et al*. and Phleboviruses [1]. Currently, there are over 520 species of sand flies recorded for the Neotropical region, with higher species richness in Brazil [2,3]. This high species richness is associated with the diversity of biomes in Brazil, which range from rainforests such as the Amazon and the Atlantic forests, to dry areas of *cerrado* and *caatinga*. Each biome has a different biogeographic history, created by events that shaped the landscape and molded the geographical distribution of animals, including sand flies and their amphibian, reptilian, avian, and mammalian hosts [4,5,6,7,8]. Vicariance, a historical event characterized by the geographical separation and isolation of a subpopulation, for example, can lead to speciation due reduced gene flow between subpopulations of the same species [9,10]. As a result of these geographical barriers, subpopulations begin to evolve independently and to accumulate genetic differences that can lead to speciation. In the event of speciation, a subpopulation loses the ability to mate successfully with other subpopulations, even if the geographical barrier is later removed. Over time, distinct morphological characters can also evolve; however, morphological differentiation tends to take longer because changes in morphological traits require changes in multiple genes [10,11]. Morphological differences among species have been the basis of taxonomy and classification for several groups of animals, including phlebotomine sand flies [2,12,13,14,15].

Currently, there are two classification systems for American sand flies. The first classification divided the New World sand fly species into three genera: *Warileya* Hertig, *Brumptomyia* França & Parrot, and *Lutzomyia* França. *Lutzomyia* was subdivided further into several subgenera and species groups [12,13]. The second classification also recognized the genera *Warileya*, *Brumptomyia* and *Lutzomyia*, but elevated several subgenera and species groups within the *Lutzomyia* genus *sensu* [12,13] to the generic rank [2,16]. This latter classification is preferred among Brazilian taxonomists because it provides a well-supported hypothesis about the evolutionary relationships among the species and genera within Phlebotominae [17,18,19,20]. Despite the additional generic ranking for some species, both sand fly classifications are based only on morphological characters. This method of morphology-based identification is limited by the inability to recognize different species that may be components of a cryptic series complex, that is, subpopulations in the early stages of speciation that have not yet evolved morphological differences. For example, the morphology of females from closely related species of sand flies belonging to *Brumptomyia*, *Evandromyia* Mangabeira, *Pressatia* Mangabeira, and *Trichophoromyia* Barretto are too similar to allow species discrimination based on morphological characteristics [21,22,23,24,25,26]. Additionally, morphology-based identifications may lead to the inappropriate grouping of subpopulations into different species because of phenotypic variation. There are several examples of intraspecific phenotypic variation among sand flies [27,28,29,30], which may prevent accurate identification of different sand fly species [31,32] Nevertheless, correct species identification is critical, particularly for species of epidemiological importance [33,34]. Therefore, the use of reliable tools for species identification to support morphology-based identification systems must be encouraged. One promising approach to the diagnosis of biological diversity is DNA barcoding, which uses a standardized fragment of DNA that is compared to a database of known sequences [35,36]. By using a 658 bp fragment from the mitochondrial gene, *cytochrome c oxidase subunit I* (*COI*), this method has facilitated the identification and discovery of new species among many taxa of insects, including butterflies, black flies and mosquitoes [33,37,38]. Previous studies have successfully utilized DNA barcoding to identify cryptic species of sand flies [39] and to validate species that were previously identified based on morphology [34,40]; however, DNA barcoding has never been applied to the identification of Brazilian sand flies. Because of high sand fly species richness in the Neotropical region, and lack of distinguishing morphological characters among females of some closely related species, we sought to test the utility of the DNA barcode approach in species identification of phlebotomine sand flies in Brazil.

## Materials and Methods

### Sand fly Collection and Morphological Identification

**Fig 1:**
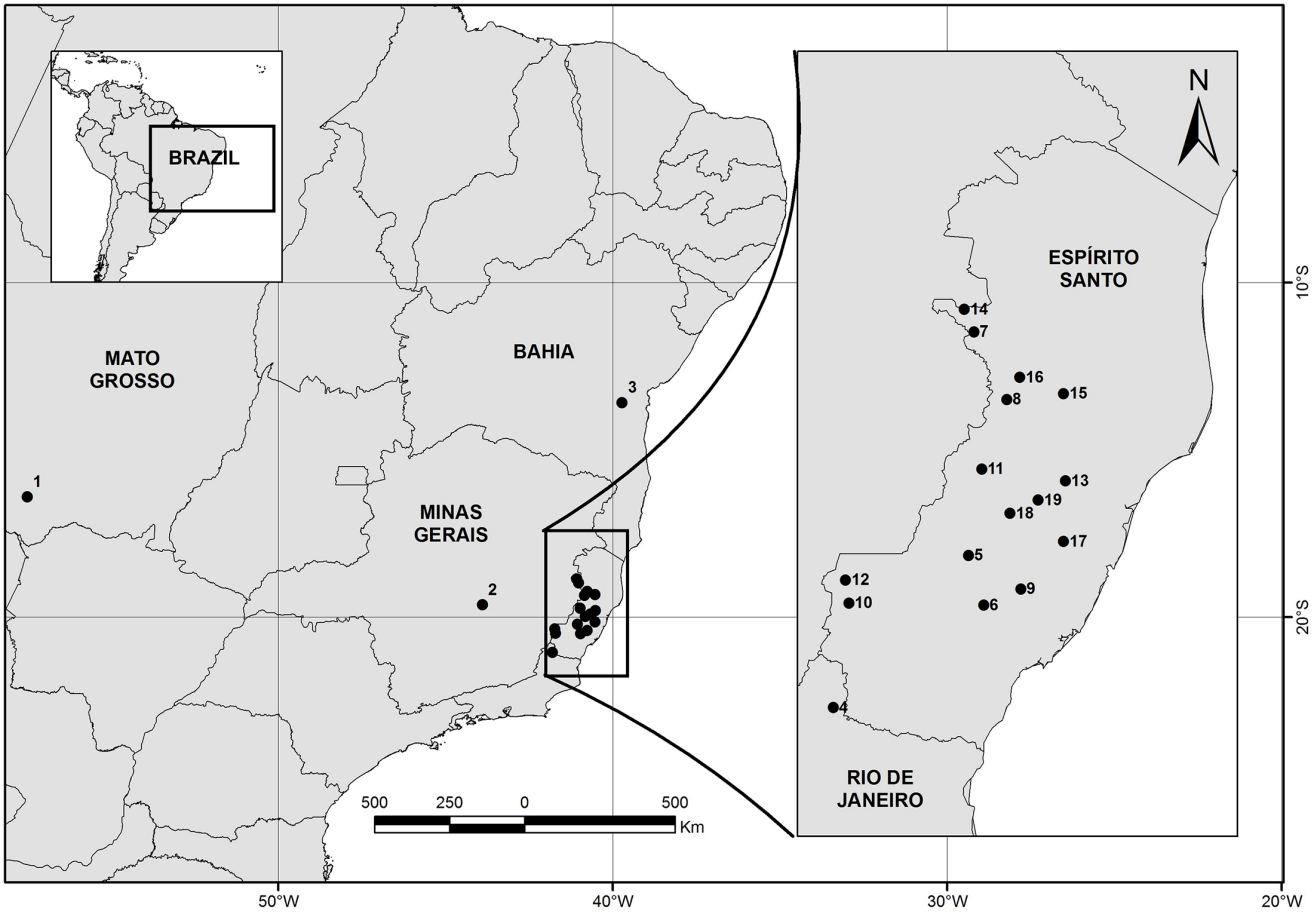
Map showing the sampling sites across the Brazilian territory. (1) Cáceres. (2) Lagoa Santa. (3) Wenceslau Guimarães. (4) Bom Jesus do Itabapoana. (5) Afonso Cláudio. (6) Alfredo Chaves. (7) Alto Rio Novo. (8) Baixo Guandu. (9) Domingos Martins. (10) Ibitirama. (11) Itaguaçu. (12) Iúna. (13) João Neiva. (14) Manenópolis. (15) Marilândia. (16) Pancas. (17) Santa Leopoldina. (18) Santa Maria de Jetibá. (19) Santa Teresa.

All the sand flies collections were performed using a permanent license to collect zoological material (process number: 32102) provide by the Instituto Brasileiro do Meio Ambiente e dos Recursos Naturais Renováveis (IBAMA). The collections performed in private areas also received a verbal permission to conduct the study on the site by the land owner. Sand flies were collected between 2011 and 2013 from 19 municipalities distributed across five Brazilian states: State of Mato Grosso: 1) Cáceres (16°24’08” S; 57°29’55” W; 286 meters at the sea level—a.s.l); State of Minas Gerais: 2) Lagoa Santa (10°45’48” S; 48°06’32” W; 740 m a.s.l.); State of Bahia: 3) Wenceslau Guimarães (13°35’04” S; 39°42’32” W; 455 m a.s.l); State of Rio de Janeiro: 4) Bom Jesus do Itabapoana (21°03’25” S; 41°47’31” W; 500 m a.s.l.); State of Espírito Santo: 5) Afonso Cláudio (20°12’53” S; 41°02’31” W; 1030 m a.s.l.), 6) Alfredo Chaves (20°29’25” S; 40°57’28” W; 1069 m a.s.l.), 7) Alto Rio Novo (18°58’35” S; 41°00’43” W; 762 m a.s.l.), 8) Baixo Guandu (19°21’04” S; 40°49’48” W; 719 m a.s.l.), 9) Domingos Martins (20°24’01” S; 40°45’11” W; 673 m a.s.l.), 10) Ibitirama (20°28’41” S; 40°42’19” W; 842 m a.s.l.), 11) Itaguaçu (19°44’13” S; 40°58’09” W; 871 m a.s.l.), 12) Iúna (20°21’02” S; 41°43’27 W; 851 m a.s.l.), 13) João Neiva (19°48’07” S; 40°30’23” W; 632 m a.s.l.), 14) Mantenópolis (18°51’09” S; 41°03’59” W; 661 m a.s.l.), 15) Marilândia (19°19’04” S; 40°31’01” W; 581 m a.s.l.), 16) Pancas (19°13’44” S; 40°45’31” W; 133 m a.s.l.), 17) Santa Leopoldina (20°08’16” S; 40°30’57 W; 51 m a.s.l.), 18) Santa Maria de Jetibá (19°58’54” S; 40°48’46” W; 844 m a.s.l.), 19) Santa Teresa (19°54’30” S; 40°39’25” W; 754 m a.s.l.). A map with these localities is shown in Fig 1.

The sand flies were captured using light traps (HP model) [41] placed within the peridomiciliary environment (represented by the domiciles and their annexes, and domestic animal shelters) and/or forest environment from 18:00 to 06:00. The insects were stored in 80% ethanol and transported to the laboratory where they were screened and separated. Sand fly legs were separated for subsequent DNA extraction, and the sand fly body was mounted on glass slides as reported by [42] for identification of the species based on morphological characters [2,43], using the classification of [14,16]. The generic names abbreviations followed [44]. Sand fly vouchers were deposited in the Coleção de Flebotomíneos (COLFLEB) of the Centro de Pesquisas René Rachou, FIOCRUZ, Belo Horizonte, state of Minas Gerais.

Species were selected based on their epidemiological importance, including *Lutzomyia longipalpis* (Lutz & Neiva) and *Lutzomyia cruzi* (Mangabeira) vectors of *Leishmania infantum* Nicolle, *Bichromomyia flaviscutellata* (Mangabeira) vector of *Leishmania* (*Leishmania) amazonensis* Lainson & Shaw and *Nyssomyia whitmani* (Antunes & Coutinho) and *Nyssomyia intermedia* (Lutz & Neiva) vectors of *Leishmania* (Viannia) *braziliensis* Vianna Other species, including *Migonemyia migonei* (França), *Pintomyia fischeri* (Pinto), *Psychodopygus ayrozai* (Barretto & Coutinho), *Psychodopygus davisi* (Root) and *Psychodopygus hirsutus* (Mangabeira), were selected because they were previously found to be infected by *Leishmania* parasites in some regions of Brazil [45,46,47,48,49,50,51,52,53,54,55].

### Genomic DNA Extraction and Analysis

Genomic DNA extraction was performed using legs of the sand fly specimen. Genomic DNA extraction was performed as reported by [56]. This is a rapid DNA extraction method for DNA amplification that comprises three steps: 1) the insect is macerated with 500 μl of extraction buffer (10mM Tris-HCl pH 8.2, 2mM EDTA, 0.2% Triton X-100) together with 1μl proteinase K (100μg/ml); 2) the sample is incubated at 50–56°C for 30 min in a OCR machine, and heated to 95°C for 10 min to inactivate the proteinase K and cooled down to 4°C; and 3) the sample is centrifuged at 15,000 *g* for 5 min at 4°C and remove the supernatant to a fresh tube. We used 20 μl of extraction buffer to macerate the legs which incubated overnight at 37°C. Polymerase chain reaction (PCR) was performed to amplify a 658 bp fragment of the *COI* gene, to a final volume of 50 μL reaction mixture containing 2 μL of genomic DNA template, 25 μL 2X Promega Go *Taq* Green™ Master Mix (Promega, Madison, WI, USA), and the primers LCO1490 (5′-GGTCAACAAATCATAAAGATATTGG-3′) and HCO2198 (5′-TAAACTTCAGGGTGACCAAAAAATCA-3′) to a final concentration of 1.0 μM [57]. The reaction cycle consisted of an initial denaturation step of 95°C for 3 min, followed by 37 cycles of 95°C for 1 min, 50°C for 1 min, 72°C for 1.5 min, and a final extension cycle 72°C for 7 min. The amplified fragments were separated by agarose (2%) gel electrophoresis and purified using Illustra GFX PCR DNA™ and Gel Band Purification Kit (GE Healthcare, Pittsburgh, PA, USA). The purified fragments were sequenced bidirectionally at Fundação Oswaldo Cruz (PDTIS/FIOCRUZ), Rio de Janeiro, Brazil with an ABI 3730 sequencer, using the same primers that were used in PCR.

### Sequence Analysis

The forward and reverse sequences for each specimen were aligned using Clustal W [58] and edited to generate a consensus sequence using BioEdit 7.0 [59]. The “Sequence Composition” tool in BOLD (Barcode of Life Database–www.boldsystems.org) was used to evaluate nucleotide content of the sequences.

Pairwise nucleotide sequence divergence was estimated between all sequences using the Kimura 2-parameter (K2P) model [60] implemented in MEGA 6.0 [61]. We used the K2P model for two reasons: (1) it makes fewer assumptions about the nature of sequence changes than more heavily parameterized models [35,62] and (2) it provides a conservative estimate of long branches than more complex models, by underestimating the number of multiple hits [63]. Levels of genetic divergence within genera and species were calculated in BOLD using the “Distance Summary” tool. Calculations of intraspecific divergence were limited to those species that were represented by at least three specimens and whose DNA sequences showed nucleotide substitutions. Female specimens that were identified only at genus level due to morphological similarities among congeners were not used in the analyses at the species level.

The K2P distances were used in MEGA 6.0 to conduct a neighbor-joining (NJ) analysis and to build a dendrogram showing the similarity among taxa, including bootstrap analysis (1000 replications). One black fly species (Diptera, Simuliidae), *Simulium metallicum s*. *l*. (GenBank number: KC015102.1), and two mosquitoes species (Diptera, Culicidae), *Ochlerotatus canadensis* (GenBank number: JX259544.1), and *Culex pipiens pallens* (GenBank number: JQ350727.1) were used as outgroups. MEGA 6.0 was also used to inspect the parsimony informative sites among closest species with little to no morphological differentiation and to highlight the fixed differences (defined as sites at which all of the sequences in one sample are different from all of the sequences in a second sample) among its sequences.

### Molecular Identification of Species

The ABGD software (Automatic Barcode Gap Discovery) [64] was used to find potential barcode gaps and for primary species delimitation. ABGD sorts sequences into hypothetical species based on the barcode gap, which can be observed whenever the intraspecific divergence is smaller than the interspecific divergence. ABGD uses a range of prior intraspecific divergences to infer a confidence limit for intraspecific divergence from within the data and then identifies and uses the barcode gap to partition the data. The inferred confidence limit and barcode gap are then recursively applied until the data is maximally partitioned. We limited the default range of intraspecific divergence between 0.001 and 0.1. Species were assigned the status of provisional species, indicated by the addition of the suffix PS to the species name, if they split into two or more distinct groups with sequence divergences between them that greater than value of the barcode gap found by ABGD. We used NJ bootstrap support values (≥80%) for the groups to corroborate the data and morphological identification to solve problems regarding species groups with low congener divergence.

### Statistical Analysis

Regression analysis was performed using STATISTICA [65] to test: (1) the relationships between sample size and intraspecific sequence divergence (mean and maximum), and (2) the relationships between sequence divergence and geographical distance within each species. The geographical distances between the collections sites were estimated using the Geographic Distance Matrix Generator version 1.2.3 [66].

## Results

**Fig 2:**
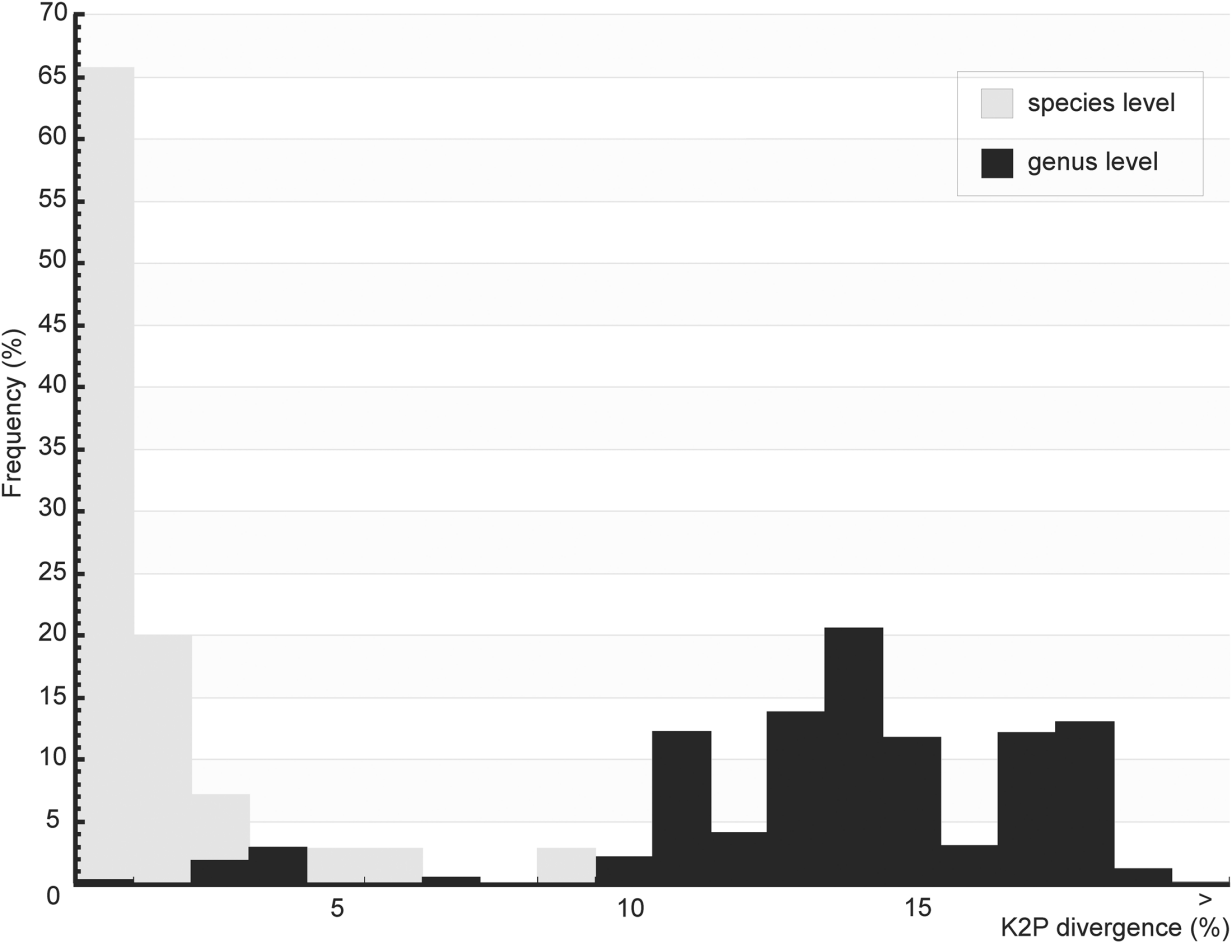
Frequency histograms of mean *COI* sequence divergences (K2P) among sand flies from Brazil. Gray bars represent mean *COI* sequence divergences (K2P) for species levels and black bars for genus levels of the taxonomic hierarchy.

**Fig 3:**
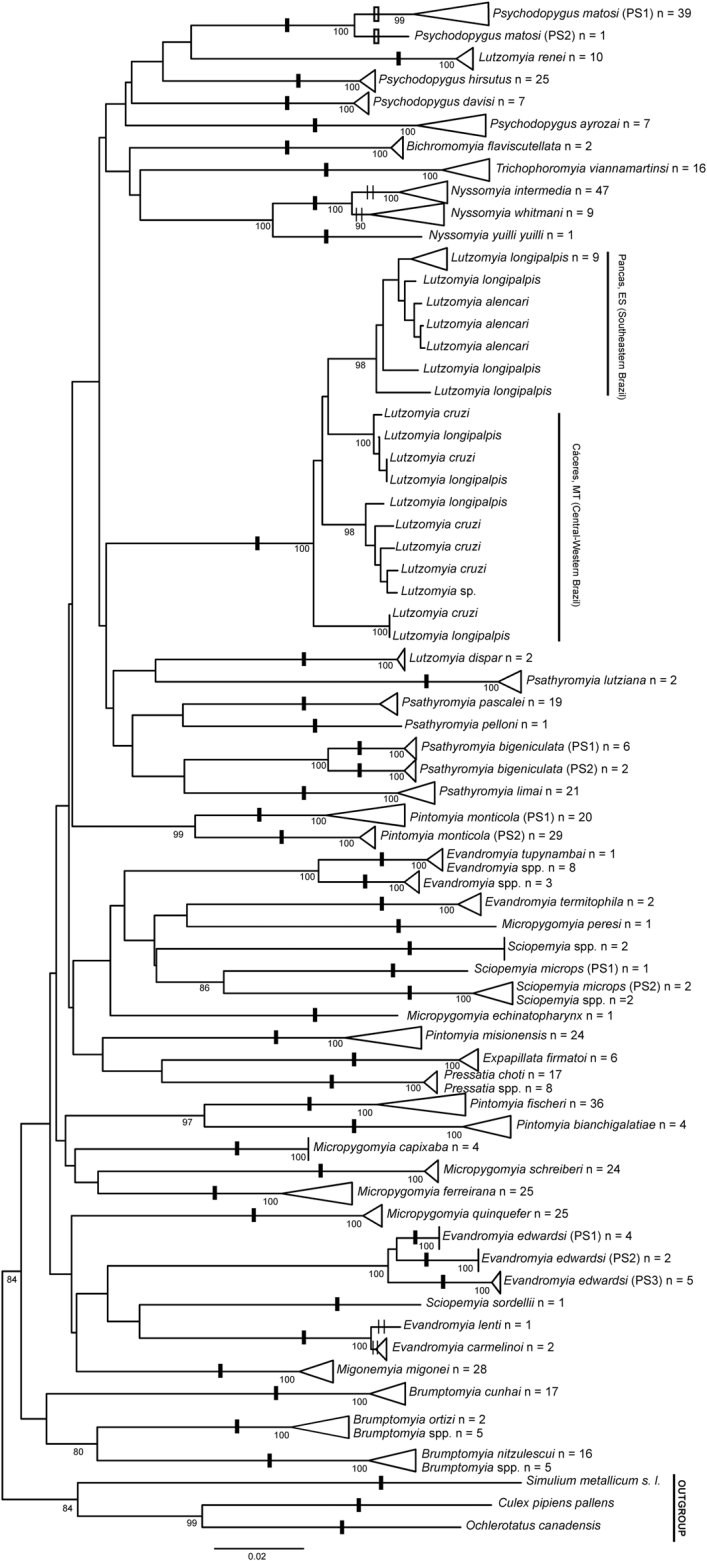
Neighbor-joining tree of *COI* sequence divergences (K2P) obtained from 567 specimens of sand flies analyzed. Numbers next to the branches indicate Bootstrap percentages ≥80%. One black dash indicates groups partitioned by ABGD using the value of 2.15%. One white dash indicates the groups partitioned by ABGD using the cut-off of 1.29%. Two thin black dashes indicated groups that were not recognized by the ABGD partitions. The number of specimens is indicated behind each species name. PS1, PS2, and PS3 represent distinct groups that are hypothesized to represent provisional species.

A total of 576 specimens of Brazilian sand flies, belonging to 47 morphologically classified species and 14 genera, were collected and subjected to barcode analysis (S1 Table). A 658 bp length of the *COI* amplicon was recovered from all 576 specimens. Sequences and original trace files are available in the “AFBR-DNA barcoding for identification of sand flies (Diptera, Psychodidae, Phlebotominae) from Brazil” project in BOLD and in GenBank (accessions numbers: KP112487—KP113062) (S2 Table). The *COI* sequences from the sampled species showed an A + T bias (mean = 0.671) relative to the C + G content (mean = 0.329), as has been found previously for arthropods [33,67]. Individual mean nucleotide content was as follows: A = 0.290, G = 0.159, C = 0.170 and T = 0.381. The mean K2P sequence distance within nominal species was 1.41%, while the mean divergence between congeners was approximately 10-fold higher (13.34%). Frequency histograms of mean *COI* sequence divergences (K2P) at both the species and genus levels of the taxonomic hierarchy are shown in Fig 2. The NJ tree, built using the K2P distances between the specimens, is shown in Fig 3 and S1 Fig.

Using ABGD, we identified nine values for potential barcode gaps (S2 Fig). We only considered barcode gaps with values of prior intraspecific divergence between 1% and 2.5% because values below 1% or above 2.5% can overestimate or underestimate, respectively, the number of species. We found two values for barcode gaps within this range: 1.29% and 2.15%. Using the value of 2.15%, the species were partitioned into 48 groups, as indicated by one black dash (Fig 3). Using the value of 1.29%, the species were partitioned into 49 groups because one of the original 48 groups [*Psychodopygus matosi* (Barretto & Zago)] was split in two groups, as indicated using one white dash (Fig 3). Seven species were not recognized by the ABGD partitioning: *Nyssomyia intermedia* was grouped with *Ny*. *whitmani*; *Ev*. *carmelinoi* (Ryan *et al*.) was grouped with *Evandromyia lenti* (Mangabeira) as indicated with two thin black dashes in the NJ tree (Fig 3); and *Lu*. *longipalpis*, which if analyzed separately, showed deep intraspecific variation that suggested it should form two groups. One of the two groups, comprising *Lu*. *longipalpis* specimens from the central-western region of Brazil, clustered with *Lu*. *cruzi* specimens, while the other group, comprising *Lu*. *longipalpis* from southeastern of Brazil, clustered with *Lutzomyia alencari* Martins, Souza & Falcão (Fig 3). Four nominal species, *Psathyromyia bigeniculata* (Floch & Abonnenc), *Evandromyia edwardsi* (Mangabeira), *Pintomyia monticola* (Costa Lima), and *Sciopemyia microps* (Mangabeira), showed deep intraspecific variation, forming two or more intraspecific barcode groups with mean divergence greater than 2.15% (Fig 3). *Psychodopygus matosi* showed moderate intraspecific variation and only formed two intraspecific barcode groups with mean divergence greater than 1.29% (Fig 3).

We found fixed differences within *Pa*. *bigeniculata*, *Ev*. *edwardsi*, *Pi*. *monticola*, and *Brumptomyia* genus and between *Evandromyia tupynambai* (Mangabeira) and *Evandromyia* spp. (S3 Fig). Because of the morphological similarities between the species, we also compared *Pa*. *bigeniculata* (PS1 and PS2) sequences to the sequences from *Pa*. *limai* (Fonseca). *Psathyromyia limai* showed 41 fixed differences in relation to both provisional species of *Pa*. *bigeniculata* (PS1 and PS2), while *Pa*. *bigeniculata* PS1 showed nine fixed differences in relation to both *Pa*. *bigeniculata* PS2 and *Pa*. *limai*. Likewise, *Pa*. *bigeniculata* PS2 showed 13 fixed differences in relation to both *Pa*. *bigeniculata* PS1 and *Pa*. *limai*. These three species still showed one fixed difference among each other that can be used as diagnostic site (421^st^ position of the *COI* sequence) (S3A Fig). The three species within the *Ev*. *edwardsi* nominal species lacked a diagnostic site among them. However, *Ev*. *edwardsi* PS1 showed eight fixed differences in relation to *Ev*. *edwardsi* PS2 and PS3, while *Ev*. *edwardsi* PS2 showed ten fixed differences in relation to *Ev*. *edwardsi* PS1 and PS3, and *Ev*. *edwardsi* PS3 showed 16 fixed differences in relation to *Ev*. *edwardsi* PS1 and PS2 (S3B Fig). The two species of *Pi*. *monticola* (PS1 and PS2) showed 36 fixed (S3C Fig). The three species of the *Brumptomyia* genus showed seven fixed differences that can be used as diagnostic sites (S3D Fig). *Evandromyia tupynambai* showed 27 fixed differences in relation to *Evandromyia* spp. (S3E Fig).

The mean intraspecific sequence divergence values (adjusted R^2^ = -0.0265, p = 0.587) (S4A Fig) and maximum intraspecific sequence divergence values (adjusted R^2^ = -0.0372, p = 0.861) (S4B Fig) were not significantly correlated to sample size. After a Bonferroni correction sequences divergences were significantly correlated to geographical distance within ten species (S4C, S4D, S4E, S4F, S4G, S4H, S4I, S4J, S4L, and S4M Fig).

## Discussion

The molecular identification of species based *COI* DNA barcoding has been useful for species recognition and the discovery of different taxa of insect vectors of parasites [33,34,39]. Here, we show that *COI* DNA barcoding is an effective tool for the discovery and recognition of sand fly species from Brazil.

First, there existed a possibility that we would not accurately detect amplification of the *COI* gene when using the sand fly genomic DNA. This concerned was based on the amplification of *COI* nuclear pseudogenes of mitochondrial origin (NUMTs) in a small number of previous DNA barcode studies using animal DNAs [68,69]. The presence of NUMT contamination is often indicated by the presence of PCR ghost bands, extra bands in restriction profiles, sequence ambiguities in polymorphic sites, or when both template strands are sequenced, by the presence of frameshift mutations, stop codons, and unexpected phylogenetic placements [68]. Additionally, in the DNA barcode studies, the amplicons generated from NUMT sequences often were shorter than the full-length barcode sequences of *COI* [69]. As shown above, all of the sequence reads from this study recovered full-length barcodes sequence, with no evidence of insertions, deletions, or stop codons, indicating the absence of NUMTs in the sequences analyzed. This finding is similar to a previous barcoding study that did not find NUMTs in Colombian sand fly species [34]. From our results, we concluded that *COI* sequences could reliably be used for barcode analysis of sand flies collected from Brazil.

The DNA barcode analyses using the automatic partitioning performed by ABGD allowed us to correctly discriminate approximately 85% (40 species) of all previously morphologically identified species (Fig 3). This percentage becomes closer to 90% when we consider that the *COI* gene can be used to discriminate between *Ny*. *intermedia* and *Ny*. *whitmani*. Although the genetic divergence between these species was too low to allow partitioning by ABGD, *Ny*. *intermedia* and *Ny*. *whitmani* both are composed of monophyletic groups with high bootstrap support values (Fig 3). Even at 90%, this identification percentage was somewhat lower than the percentages found in DNA barcode studies of other sand fly fauna. The identification percentages from studies using sand flies from Colombia and India, for example, were close to 100% [34,39]. Nevertheless, a number of DNA barcode studies in which specimen for each species were collected from several localities showed discrimination percentages similar to those reported here [33,70]. Differences between the species discrimination percentages of sand flies from Brazil and others regions may be related to sampling effects. In the study of sand flies from Colombia, 36 species were collected from different geographic localities and analyzed, 31 of which included the analysis of more than one specimen; however, only ten of these 31 species were sampled from more than one locality. Two of these ten species, *Lutzomyia gomezi* (Nitzulescu) and *Psychodopygus panamensis* Shannon, showed the maximum intraspecific *COI* sequence divergence within one species [34]. This result suggests that the increase in sampling localities for each species could influence their discrimination percentages. Our data supports this possibility, since ABGD accurately delimited between species collected from the same locality. In the study of sand flies from India, although the species were all collected from multiple localities, the high percentage of discrimination between the species may be explained by the small number of species analyzed [39]. The genetic distances between species also can be larger if some species are missing from the assemblage and, consequently, the delimitation becomes easier [71]. This raised concerns regarding the effect of sample size and geographical scale of sampling in the *COI* sequence divergence (intraspecific genetic variation and interspecific genetic divergence to congeners) for sand flies from Brazil.

A global study using diving beetles showed that both intraspecific genetic variation and interspecific genetic divergence to congenerics are correlated to the geographical scale of sampling [71]. This correlation is supported by a number of theories and concepts, including those of isolation by distance and distance decay [72,73]. Therefore, it is expected that a species sampled from a wide geographical coverage will show greater genetic variation than if the variation was estimated in a species sampled from a single smaller region [71]. As predicted, we found that intraspecific sequence divergence (K2P) in *COI* was significantly correlated to geographical distance (S4 Fig) within several, but not all, of the species analyzed. Only *Pa*. *bigeniculata* (PS1 from Santa Leopoldina, state of Espírito Santo; and PS2 from Cáceres, state of Mato Grosso) and *Sc*. *microps* (PS1 from Iúna, state of Espírito Santo; and PS2 from Pancas, state of Espírito Santo) were clustered according to geographical distance. From these samples, we were able to infer that the barcoding gene reliably discriminates between the nominal species, even with correlation between the intraspecific sequence divergence (K2P) at *COI* and the geographical distance within some species.

The effects of geographical scale on sampling in the *COI* sequence divergence has been proposed to occur because the distance of one species to the closest morphologically different species significantly decreases with increasing geographical scale of sampling [71]; however, our data did not corroborate this hypothesis. Though *Lu*. *longipalpis*, *Lu*. *cruzi* and *Lu*. *alencari* are closely related species [2,19,74], for example, specimens from these three species of the same geographical region showed less sequence divergence than the closest morphologically different species from others regions (Fig 3). *Lutzomyia longipalpis* specimens from Pancas, state of Espírito Santo, were closely related to *Lu*. *alencari* specimens from the same region, and *Lu*. *longipalpis* specimens from Cáceres, state of Mato Grosso clustered with *Lu*. *cruzi* specimens from the same region (Fig 3). Despite the morphological differences between these species, this low level of sequence divergence, suggests that *Lu*. *alencari* also belongs to the *Lu*. *longipalpis* complex. This possibility is further supported by the presence of a spot in the abdominal tergite of both species, which appears to be a synapomorphy of the *Lu*. *longipalpis* complex [19]. The low sequence divergence found between these sympatric species can be the result of introgression [75,76], a common phenomenon among closely species that tends to occur much more readily in the mitochondrial DNA than in the nuclear DNA [77,78].

Introgression can also explain the low divergence found among *Ny*. *intermedia* and *Ny*. *whitmani* (Fig 3). The evidence of introgression among these species is corroborated by studies using mitochondrial DNA (mtDNA) and using nuclear DNA [79,80,81]. Introgression of mtDNA was detected in studies analyzing *cytochrome b* gene from females that morphologically were identified as *Ny*. *intermedia* specimens from Viana, state of Espírito Santo, but showed mtDNA haplotypes from *Ny*. *whitmani* [79]. Likewise, introgression of nuclear DNA was detected by studies analyzing the *period* gene in order to differentiate species of the *Ny*. *intermedia* complex. This latter analysis indicated a low level of divergence, high similarities among sympatric sequences, and the presence of shared haplotypes between *Nyssomyia* species specimens from the locality of Posse, state of the Rio de Janeiro [80,81]. It is hard to distinguish introgression from the persistence of ancestral polymorphisms, but the introgression hypothesis has been the preferred model based on data analysis which suggested that *Ny*. *intermedia* and *Ny*. *whitmani* might be exchanging alleles [80,81].

Unlike the results for *Ny*. *intermedia* and *Ny*. *whitmani*, the bootstrap values did not support the clades of *Ev*. *carmelinoi* and *Ev*. *lenti*, and ABGD was not able to discriminate between them (Fig 3), indicating very low sequence divergence between these species. These two species are very similar morphologically, but can be differentiated by the male genital filamental tips (genital filament tips arrow-like and elongate 1/8^th^ the length of the genital filaments for *Ev*. *lenti*; and genital filaments scrolled less than 1/20^th^ the length of genital filaments for *Ev*. *carmelinoi*) and differences in the spermathecae of females (ratio of the widths of common/individual ducts is 1.5-fold higher in *Ev*. *lenti* than in *Ev*. *carmelinoi*) (See Figures 4 (1–2) and 5 (1–2) from [82]). Unfortunately, we were only able to evaluate a small number of specimens from these two species, and the sequence divergence among them (0.1% approximately) was lower than the mean of intraspecific *COI* sequence divergence within almost of all the species (S1 Table). Nonetheless, we expected that collecting the species from well separated sites (*Ev*. *carmelinoi* from Cáceres, state of Mato Grosso and *Ev*. *lenti* from Pancas, state of Espírito Santo), would favor reduction of the gene flow, thereby increasing sequence divergence. Furthermore, this similarity of sequences cannot be explained by the phenomenon of decreasing interspecific divergence with increasing geographical scale of sampling, because the geographical distance among the areas (S3 Table) is not enough to achieve such similarity (See Figure 4 from [71]). If these two nominal species really represent two different taxa, the only likely explanation is that strong introgressive hybridization occurs between *Ev carmelinoi* and *Ev*. *lenti* in Cáceres, where these two species are sympatric [83]. It should be noted, however, that only *Ev*. *lenti* has been reported in Pancas. Molecular and morphological revision of the species group using a larger number of specimens from several localities throughout its distribution range is required to resolve this discrepancy.

While our study was in progress, the shannoni group of the genus *Psathyromyia* was revised [43], including species previously reported for the state of Espírito Santo as *Psathyromyia shannoni* (Dyar) and *Psathyromyia pestanai* (Barretto & Coutinho) [84,85,86]. From the revision, two species of the shannoni complex, *Pa*. *bigeniculata* and *Pa*. *limai*, were resurrected from the synonymy of *Pa*. *shannoni*, while a third species, *Pa*. *pestanai*, was proposed to be a junior synonym of *Pa*. *limai* on the basis of morphological characters. Therefore, we reexamined all slides of the specimens morphologically identified as *Pa*. *shannoni* from states of Espírito Santo state and Mato Grosso and found them to be *Pa*. *bigeniculata* from Cáceres, Mato Grosso and from Santa Leopoldina, state of Espírito Santo. We also found *Pa*. *limai* from the remaining areas of the state of Espírito Santo. Corroborating the results of the morphological revision, the DNA barcode discriminated between *Pa*. *limai* and *Pa*. *bigeniculata*, showing a high mean sequence divergence (10%) between them (Fig 3). It is noteworthy that the DNA barcode analysis from our study split the specimens of *Pa*. *bigeniculata* into two groups (PS1 from state of Espírito Santo and PS2 from Cáceres from state of Mato Grosso) that represent putative species within the *Pa*. *bigeniculata* complex (Fig 3). These groups had a high number of fixed differences among them, including a diagnostic site (421^st^ position of the 658 bp fragment of *COI* gene) that enabled identification of *Pa*. *bigeniculata* (PS1 and PS2) and *Pa*. *limai* (S3A Fig). Based on the distribution of the analyzed specimens, *Pa*. *bigeniculata* PS1 represented a coastal species (Atlantic Forest) and *Pa*. *bigeniculata* PS2 represented an interior species (*cerrado*). Besides supporting the resurrection of two species within the shannoni group that are morphologically similar, the DNA barcode also provided a reliable method to discriminate among them, corroborating the utility of the DNA barcode for sand fly identification.

Our analyses also suggested that three other nominal species belongs to cryptic species complex: *Ev*. *edwardsi*, *Pi*. *monticola*, and *Sc*. *microps*. *Evandromyia edwardsi* was split into three species (PS1, PS2, and PS3) (Fig 3), each with fixed differences in relation to the other species (S3B Fig), but without a diagnostic site. The fixed differences set can be used to differentiate between these three species within the *Ev*. *edwardsi* complex. *Evandromyia edwardsi* does not morphologically resemble other sand fly species, and therefore, can be easily identified based on the male paramere and shape of the spermathecae of the female. This morphological distinction and the low number of specimens collected throughout the distribution range of *Ev*. *edwardsi* might have contributed to the failure to recognize slight morphological differences among them. We also failed to find morphological differences within the *Pi*. *monticola* complex; however, fixed differences in the *COI* sequences resulted in diagnostic sites useful for distinguishing the two species (PS1 and PS2), which were split with substantial mean sequence divergence among them (8.1%) (Fig 3, S3C Fig). We found slight morphological differences between the females of the two species (PS1 and PS2) within *Sc*. *microps*, which also showed high mean sequence divergence among them (11.9%). *Sciopemyia microps* PS1 was composed of one female specimen, while *Sc*. *microps* PS2 was composed of two male and two female specimens, identified as *Sciopemyia* sp. The females grouped as *Sc*. *microps* PS2 were morphologically identified as *Sciopemyia* sp. because, due to slide preparation, it was not possible to exam the spermathecae, and because the cibarium (horizontal teeth less-development) was very different from the morphological description of the females of *Sc*. *microps* (horizontal teeth well-developed). Unlike the *Sc*. *microps* PS2 females, the female of *Sc*. *microps* PS1 was morphologically similar to the description of *Sc*. *microps* [87], with well-developed horizontal teeth in the cibarium. Two other female specimens (Sc_sp255f and Sc_sp299f, S1 Fig) were also identified as *Sciopemyia* sp. and were partitioned into a third species based on our DNA barcode analyses (Fig 3). This result supports a previous sand fly survey of the area, which identified these specimens as belonging to *Sc*. aff. *microps* rather than *Sc*. *microps* [24] because of differences in the diameter of the female spermathecae ducts (AL Ferreira, *personal communication*). These slight morphological differences are difficult to detect and require considerable technical skills. Therefore, DNA barcode analysis is a useful alternative for discriminating among specimens with small morphological differences.

Finally, the DNA barcode analysis was able to associate morphologically indistinguishable females with males of the genera *Brumptomyia*, *Evandromyia*, and *Pressatia*. Based on the male morphology, three species of *Brumptomyia* [*Brumptomyia cunhai* Mangabeira, *Brumptomyia ortizi* Martins, Silva & Falcão, and *Brumptomyia nitzulescui* (Costa Lima)] were identified and all the females were identified as *Brumptomyia* sp. The specimens of *Br*. *cunhai* were collected from a sand fly survey from Pancas, Espírito Santo and females from this locality were not analyzed. Unfortunately, this species was not included in the sand fly survey from that area because, at that time, *Br*. *cunhai* specimens were misidentified as *Br*. *nitzulescui* [24]. Since the initial survey, we performed a morphological revision of the specimens based on a preliminary barcode analyses which indicated two groups of *Br*. *nitzulescui* (one from Pancas and others from others areas). Indeed, we find that the group from Pancas was composed of specimens of *Br*. *cunhai*. This result highlights the importance of integrative approaches based on morphology and molecular biology to reach accurate results with sand fly identification. In our study, *Br*. *cunhai* showed 46 fixed differences in relation to *Br*. *ortizi* and *Br*. *nitzulescui*, including seven fixed differences that can be used as diagnostic sites among these three species. Besides the seven diagnostic sites, *Br*. *ortizi* showed 26 fixed differences in relation to *Br*. *cunhai* and *Br*. *nitzulescui* (S3 Fig). Five of the ten females identified as *Brumptomyia* sp. were grouped with the *Br*. *ortizi* males, sharing its fixed differences in relation to others *Brumptomyia* species. The automatic partitioning by ABGD also indicated that these females belong to *Br*. *ortizi* taxon. In addition to the similarities between the *COI* gene sequences, the specimens were collected at the same locality, which further supports that they belong to the same taxon. This finding is very interesting and underscores the suitability of DNA barcoding for describing *Br*. *ortizi* females, since females of *Br*. *ortizi* have not been previously described [2] because it is morphologically similar to other females of the *Brumptomyia* genus. Females of *Br*. *nitzulescui* can be morphologically differentiated from five others *Brumptomyia* species, but when sympatric with others species [88], the differentiation is difficult to achieve. Our DNA barcode analysis clustered five *Brumptomyia* females specimens within the *Br*. *nitzulescui* clade, which showed a high number of fixed differences and, consequently, high sequence divergence in relation to others *Brumptomyia* species. As noted for *Br*. *ortizi*, this result highlights the utility of the DNA barcode tool for sand fly species identification and its potential for female and male species association, even for species without formal morphological description of both sexes. Our DNA barcode analysis also indicated an association between the males and the females of the *Evandromyia* genus. *Evandromyia tupynambai* was described for both sexes, but when found to be sympatric with *Evandromyia callipyga* (Martins & Silva) or *Evandromyia costalimai* (Mangabeira) the females of these three species cannot be distinguished. We did not analyze the males of *Ev*. *callipyga* or *Ev*. *costalimai*; however our data suggest that the barcode gene can discriminate among these species since the females identified as *Evandromyia* sp. were clustered in two different groups with high sequence divergence and fixed differences among them (Fig 3). The first group belonged to *Ev*. *tupynambai* and was composed of females. The second group of three females belonged to either to *Ev*. *callipyga* or *Ev*. *costalimai*, but could not be distinguished further because of morphological similarities with the females of *Ev*. *tupynambai* of the first group and because we did not analyze DNA barcode from their males. The high genetic difference among the females further underscores the usefulness of the DNA barcode method for associating males with females of a species. Finally, we provided a DNA barcode profile for the females of the *Pressatia* genus, which indicated that these females belong to *Pressatia choti* (Floch & Abonnenc) (Fig 3). This result is supported by the absence of other *Pressatia* species in the region where our specimens were collected [24].

In conclusion, except in cases of species that have undergone introgressive hybridization, DNA barcoding allowed discrimination of sand fly species from Brazil and the discovery of species within putative sand fly species complex. This method also reliably detected sand fly species misidentification and allowed the association between males and females among species that are morphologically similar. Finally, this study highlights the importance of utilizing integrative approaches for sand fly species identification in order to achieve accurate and reliable results.

## Supporting Information

S1 Fig A neighbor-joining tree of *COI* sequence divergences (K2P) in 47 sand fly species from Brazil. Only bootstrap values higher than 80 are shown. Each tip label in the tree contains the sand fly species name abbreviation (five words), the sample ID (number) and sex of the specimen (f = female or m = male). The sand fly species names were abbreviated as follows: Bi_fla = *Bichromomyia flaviscutellata;* Br_nit = *Brumptomyia nitzulescui*; Br_cun = *Brumptomyia cunhai*; Br_ort = *Brumptomyia ortizi*; Br_sp = *Brumptomyia* spp.; Ev_car = *Evandromyia carmelinoi*; Ev_edw = *Evandromyia edwardsi*; Ev_len = *Evandromyia lenti*; Ev_sp = *Evandromyia* spp.; Ev_ter = *Evandromyia termitophila*; Ev_tup = *Evandromyia tupynambai*; Ex_fir = *Expapillata firmatoi*; Lu_ale = *Lutzomyia alencari*; Lu_cru = *Lutzomyia cruzi*; Lu_dis = *Lutzomyia dispar*; Lu_ren = *Lutzomyia renei*; Lu_sp = *Lutzomyia* sp.; Mi_cap = *Micropygomyia capixaba*; Mi_ech = *Micropygomyia echinatopharynx*; Mi_fer = *Micropygomyia ferreirana*; Mi_per = *Micropygomyia peresi*; Mi_qui = *Micropygomyia quinquefer*; Mi_sch = *Micropygomyia schreiberi*; Mg_mig = *Migonemyia migonei*; Ny_int = *Nyssomyia intermedia*; Ny_whi = *Nyssomyia whitmani*; Ny_yui = *Nyssomyia yuilli yuilli*; Pi_bia = *Pintomyia bianchigalatiae*; Pi_fis = *Pintomyia fischeri*; Pi_mis = *Pintomyia misionensis*; Pi_mon = *Pintomyia monticola*; Pr_cho = *Pressatia choti*; Pr_sp = *Pressatia* spp.; Pa_big = *Psathyromyia bigeniculata*; Pa_lim = *Psathyromyia limai*; Pa_lut = *Psathyromyia lutziana*; Pa_pas = *Psathyromyia pascalei*; Pa_pel = *Psathyromyia pelloni*; Ps_ayr = *Psychodopygus ayrozai*; Ps_dav = *Psychodopygus davisi*; Ps_hir = *Psychodopygus hirsutus*; Ps_mat = *Psychodopygus matosi*; Sc_mic = *Sciopemyia microps*; Sc_sor = *Sciopemyia sordellii*; Sc_sp = *Sciopemyia* spp.; Th_via = *Trichophoromyia viannamartinsi*.
(PDF)

S2 Fig Number of groups among the 576 specimens of sand flies from Brazil based on the values of prior intraspecific divergence found by the Automatic Barcode Gap Discovery (ABGD) software as potential barcode gaps using a range of 0.001 to 0.1 for prior intraspecific divergence. (PDF)

S3 Fig Parsimony informative sites from a fragment of the 658 bp of the *cytochrome c oxidase subunit I* (*COI*) gene among closest related species of sand flies from Brazil. A) *Psathyromyia bigeniculata* (PS1 and PS2); B) *Evandromyia edwardsi* (PS1, PS2, and PS3); C) *Pintomyia monticola* (PS1 and PS2); D) *Brumtomyia* genus (*Brumptomyia cunhai*, *Brumptomyia ortizi*, and *Brumptomyia nitzulescui*); and E) *Evandromyia tupynambai* and *Evandromyia* spp. (yellow = fixed differences; red = diagnostic sites).
(PDF)

S4 Fig Correlation analyses showing the relationship between mean intraspecific sequence divergence (K2P) at *COI* and sample size (A), the relationship between maximum interspecific sequence divergence and sample size (B), and the relationship between sequence divergence and geographical distance among each species collected from more than one site (C-M). (PDF)

S1 Table Table showing the collections sites and number and sex of sand fly specimens for each analyzed species used to create the DNA barcoding tree in the Fig 2. Maximum and mean intraspecific values of genetic divergence (Kimura 2-parameter pairwise distances) are shown. As implemented by the Barcode of Life Database (BOLD), values are given only to species represented by three or more individuals and showing at least one nucleotide substitution. Nominal species marked with one asterisk showed distinct intraspecific lineages suggesting cryptic species complexes. Classification follows [14,16] and the generic abbreviations follow [44].
(PDF)

S2 Table Phlebotominae taxa sampled, museum specimen number, BOLD and GenBank accession numbers. (PDF)

S3 Table Matrix of geographical distance between the sampling localities estimated using the Geographic Distance Matrix Generator version 1.2.3. All the distances were estimated in kilometers. (BA = state of Bahia; ES = state of Espírito Santo; MG = state of Minas Gerais; MT = Mato Grosso; RJ = state of Rio de Janeiro).
(PDF)
