# Supplementary material for: DNA Barcoding of Neotropical Sand Flies (Diptera, Psychodidae, Phlebotominae): Species Identification and Discovery within Brazil
Source: PLoS One. 2015 Oct 27;10(10):e0140636. doi: 10.1371/journal.pone.0140636 (PMC4624639; doi:10.1371/journal.pone.0140636)
Supplement: S1 Fig — Each tip label in the tree contains the sand fly species name abbreviation (five words), the sample ID (number) and sex of the specimen (f = female or m = male). The sand fly species names were abbreviated as follows: Bi_fla = Bichromomyia flaviscutellata; Br_nit = Brumptomyia nitzulescui; Br_cun = Brumptomyia cunhai; Br_ort = Brumptomyia ortizi; Br_sp = Brumptomyia spp.; Ev_car = Evandromyia carmelinoi; Ev_edw = Evandromyia edwardsi; Ev_len = Evandromyia lenti; Ev_sp = Evandromyia spp.; Ev_ter = Evandromyia termitophila; Ev_tup = Evandromyia tupynambai; Ex_fir = Expapillata firmatoi; Lu_ale = Lutzomyia alencari; Lu_cru = Lutzomyia cruzi; Lu_dis = Lutzomyia dispar; Lu_ren = Lutzomyia renei; Lu_sp = Lutzomyia sp.; Mi_cap = Micropygomyia capixaba; Mi_ech = Micropygomyia echinatopharynx; Mi_fer = Micropygomyia ferreirana; Mi_per = Micropygomyia peresi; Mi_qui = Micropygomyia quinquefer; Mi_sch = Micropygomyia schreiberi; Mg_mig = Migonemyia migonei; Ny_int = Nyssomyia intermedia; Ny_whi = Nyssomyia whitmani; Ny_yui = Nyssomyia yuilli yuilli; Pi_bia = Pintomyia bianchigalatiae; Pi_fis = Pintomyia fischeri; Pi_mis = Pintomyia misionensis; Pi_mon = Pintomyia monticola; Pr_cho = Pressatia choti; Pr_sp = Pressatia spp.; Pa_big = Psathyromyia bigeniculata; Pa_lim = Psathyromyia limai; Pa_lut = Psathyromyia lutziana; Pa_pas = Psathyromyia pascalei; Pa_pel = Psathyromyia pelloni; Ps_ayr = Psychodopygus ayrozai; Ps_dav = Psychodopygus davisi; Ps_hir = Psychodopygus hirsutus; Ps_mat = Psychodopygus matosi; Sc_mic = Sciopemyia microps; Sc_sor = Sciopemyia sordellii; Sc_sp = Sciopemyia spp.; Th_via = Trichophoromyia viannamartinsi. (PDF) [file pone.0140636.s001.pdf]

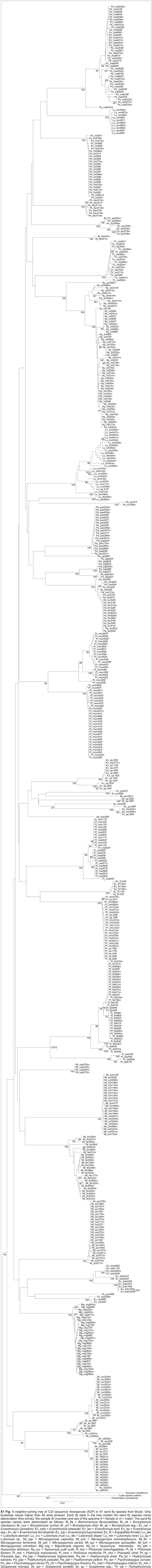

S1 Fig. A neighbor-joining tree of COI sequence divergences (K2P) in 47 sand fly species from Brazil. Only bootstrap values higher than 80 were showed. Each tip label in the tree contain the sand fly species name abbreviation (five words), the sample ID (number) and sex of the specime (f = female or m = male). The sand fly species names were abbreviated as follows: Bi_fla = *Bichromomyia flaviscutellata;* Br_nit = *Brumptomyia nitzulescui*; Br_cun = *Brumptomyia cunhai*; Br_ort = *Brumptomyia ortizi*; Br_sp = *Brumptomyia* spp.; Ev_car = *Evandromyia carmelinoi*; Ev_edw = *Evandromyia edwardsi*; Ev_len = *Evandromyia lenti*; Ev_sp = *Evandromyia* spp.; Ev_ter = *Evandromyia termitophila*; Ev_tup = *Evandromyia tupynambai*; Ex_fir = *Expapillata firmatoi*; Lu_ale = *Lutzomyia alencari*; Lu_cru = *Lutzomyia cruzi*; Lu_dis = *Lutzomyia dispar*; Lu_ren = *Lutzomyia renei*; Lu_sp = *Lutzomyia* sp.; Mi_cap = *Micropygomyia capixaba*; Mi_ech = *Micropygomyia echinatopharynx*; Mi_fer = *Micropygomyia ferreirana*; Mi_per = *Micropygomyia peresi*; Mi_qui = *Micropygomyia quinquefer*; Mi_sch = *Micropygomyia schreiberi*; Mg_mig = *Migonemyia migonei*; Ny_int = *Nyssomyia intermedia*; Ny_whi = *Nyssomyia whitmani*; Ny_yui = *Nyssomyia yuilli yuilli*; Pi_bia = *Pintomyia bianchigalatiae*; Pi_fis = *Pintomyia fischeri*; Pi_mis = *Pintomyia misionensis*; Pi_mon = *Pintomyia monticola*; Pr_cho = *Pressatia choti*; Pr_sp = *Pressatia* spp.; Pa_big = *Psathyromyia bigeniculata*; Pa_lim = *Psathyromyia limai*; Pa_lut = *Psathyromyia lutziana*; Pa_pas = *Psathyromyia pascalei*; Pa_pel = *Psathyromyia pelloni*; Ps_ayr = *Psychodopygus ayrozai*; Ps_dav = *Psychodopygus davisi*; Ps_hir = *Psychodopygus hirsutus*; Ps_mat = *Psychodopygus matosi*; Sc_mic = *Sciopemyia microps*; Sc_sor = *Sciopemyia sordellii*; Sc_sp = *Sciopemyia* spp.; Th_via = *Trichophoromyia viannamartinsi*.
