# Supplementary figures and images for: DNA Barcoding of Neotropical Sand Flies (Diptera, Psychodidae, Phlebotominae): Species Identification and Discovery within Brazil
Source: PLoS One. 2015 Oct 27;10(10):e0140636. doi: 10.1371/journal.pone.0140636 (PMC4624639; doi:10.1371/journal.pone.0140636)

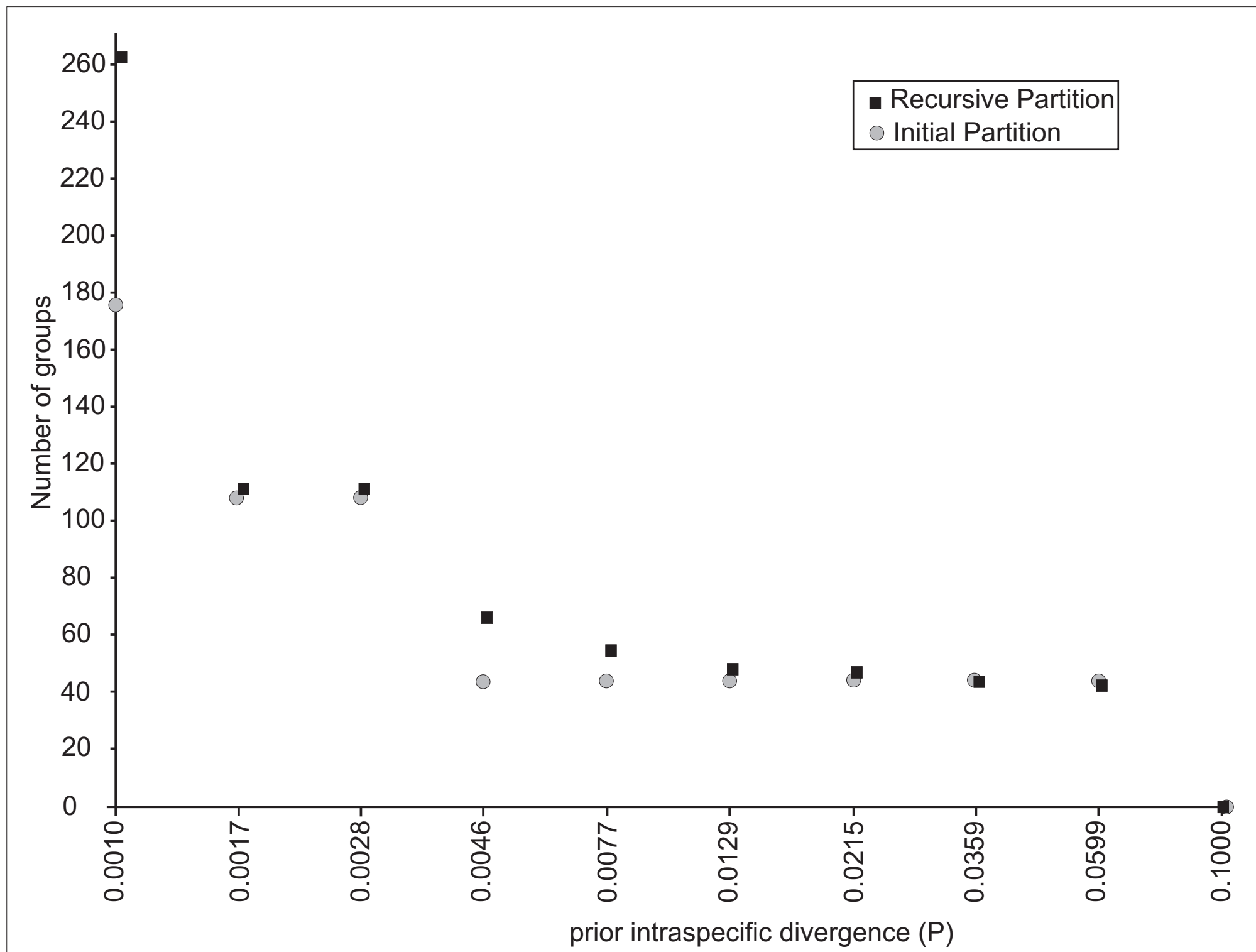

Supplement: S2 Fig — (PDF) [file pone.0140636.s002.pdf]
