# Supplementary material for: DNA Barcoding of Neotropical Sand Flies (Diptera, Psychodidae, Phlebotominae): Species Identification and Discovery within Brazil
Source: PLoS One. 2015 Oct 27;10(10):e0140636. doi: 10.1371/journal.pone.0140636 (PMC4624639; doi:10.1371/journal.pone.0140636)
Supplement: S3 Fig — A) Psathyromyia bigeniculata (PS1 and PS2); B) Evandromyia edwardsi (PS1, PS2, and PS3); C) Pintomyia monticola (PS1 and PS2); D) Brumtomyia genus (Brumptomyia cunhai, Brumptomyia ortizi, and Brumptomyia nitzulescui); and E) Evandromyia tupynambai and Evandromyia spp. (yellow = fixed differences; red = diagnostic sites). (PDF) [file pone.0140636.s003.pdf]

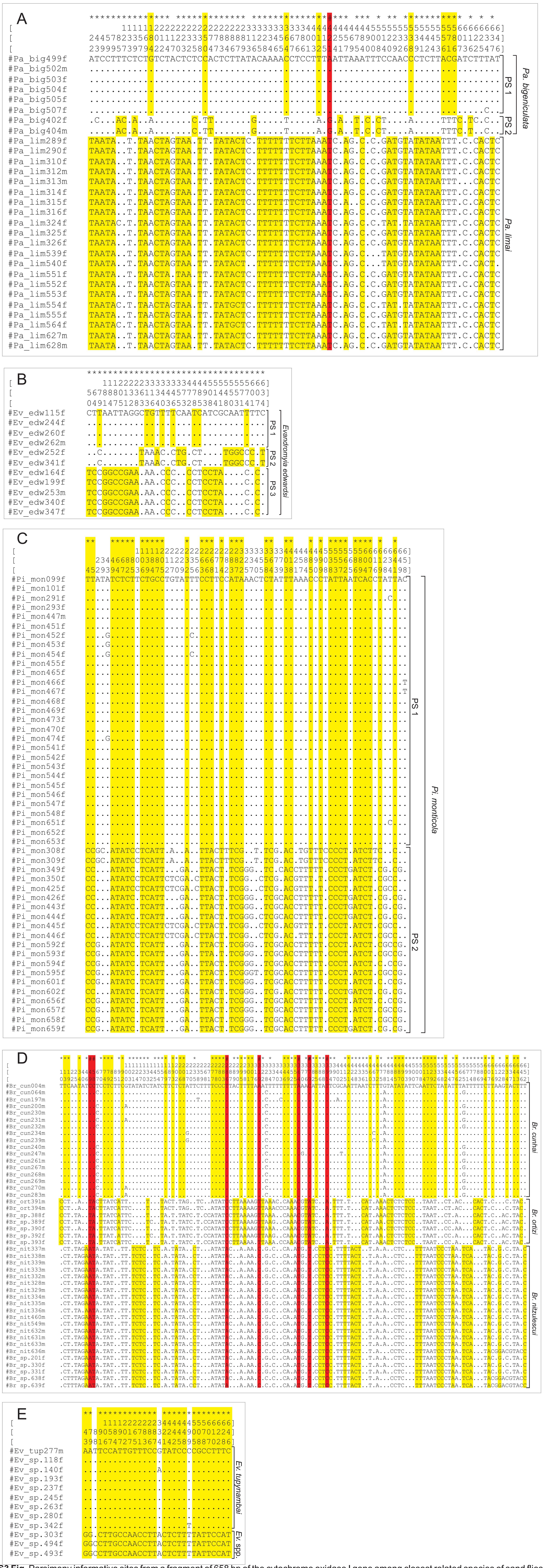

S3 Fig. Parsimony informative sites from a fragment of 658 bp of the cytochrome oxidase I gene among closest related species of sand flies from Brazil. A) *Psathyromyia bigeniculata* (PS 1 and PS 2); B) *Evandromyia edwardsi* (PS 1, PS 2, and PS 3); C) *Pintomyia monticola* (PS 1 and PS 2); D) *Brumtomyia genus* (*Brumptomyia cunhai*, *Brumptomyia ortizi*, and *Brumptomyia nitzulescui*); and E) *Evandromyia tupynambai* and *Evandromyia* spp. (yellow = fixed differences; red = diagnostic sites).
