# Supplementary material for: DNA Barcoding of Neotropical Sand Flies (Diptera, Psychodidae, Phlebotominae): Species Identification and Discovery within Brazil
Source: PLoS One. 2015 Oct 27;10(10):e0140636. doi: 10.1371/journal.pone.0140636 (PMC4624639; doi:10.1371/journal.pone.0140636)

Graph	Species	Adjusted R^2	p
C	*Lutzomyia longipalpis*	0.4532	< 0.001
D	*Micropygomyia ferreirana*	0.1950	< 0.001
E	*Micropygomyia schreiberi*	0.0677	< 0.001
F	*Migonemyia migonei*	0.0575	< 0.001
G	*Nyssomyia intermedia*	0.0236	< 0.001
H	*Pintomyia fischeri*	0.0367	< 0.001
I	*Pintomyia misionensis*	0.3576	< 0.001
J	*Pintomyia monticola*	0.3000	< 0.001
L	*Psathyromyia bigeniculata*	0.9885	< 0.001
M	*Psychodopygus matosi*	0.0145	< 0.001

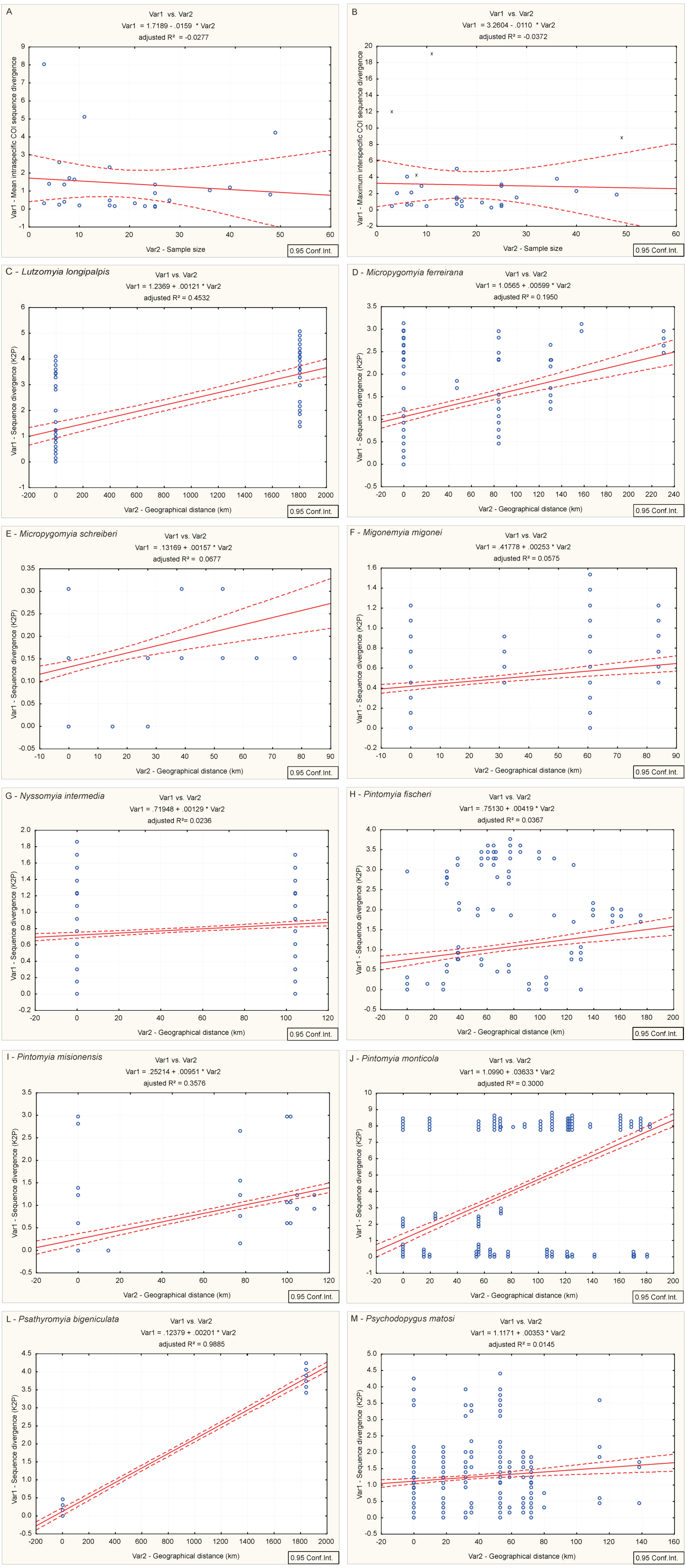

Supplement: S4 Fig — (PDF) [file pone.0140636.s004.pdf]
