# Supplementary material for: DNA Barcoding of Neotropical Sand Flies (Diptera, Psychodidae, Phlebotominae): Species Identification and Discovery within Brazil
Source: PLoS One. 2015 Oct 27;10(10):e0140636. doi: 10.1371/journal.pone.0140636 (PMC4624639; doi:10.1371/journal.pone.0140636)
Supplement: S1 Table — Maximum and mean intraspecific values of genetic divergence (Kimura 2-parameter pairwise distances) are shown. As implemented by the Barcode of Life Database (BOLD), values are given only to species represented by three or more individuals and showing at least one nucleotide substitution. Nominal species marked with one asterisk showed distinct intraspecific lineages suggesting cryptic species complexes. Classification follows [14,16] and the generic abbreviations follow [44]. (PDF) [file pone.0140636.s005.pdf]

S1 Table. Table showing the collections sites and number and sex of sand fly specimens for each analyzed species used to create the DNA barcoding tree in the Figure 2. Maximum and mean intraspecific values of genetic divergence (Kimura 2-parameter pairwise distances) are shown. As implemented by the Barcode of Life Database (BOLD), values are given only to species represented by three or more individuals and showing at least one nucleotide substitution. Nominal species marked with one asterisk showed distinct intraspecific lineages suggesting cryptic species complexes. Classification follows Galati (1995, 2003b) and the generic abbreviations follow Marcondes (2007).

**Genus**<br>Species	Locality, State	♂	♀	% divergence<br>[max. (mean)]
Bichromomyia				-/-
Bichromomyia flaviscutellata	Santa Teresa, ES	2		-/-
Brumptomyia				16.12 (12.5)
Brumptomyia cunhai	Pancas, ES	17		1.08 (0.39)
Brumptomyia nitzulescui	Alto Rio Novo, ES	1		1.54 (0.28)
	Domingos Martins, ES	4		
	Santa Maria de Jetibá, ES	11		
Brumptomyia ortizi	Bom Jesus do Itabapoana, RJ	2		-/-
Brumptomyia spp.	Bom Jesus do Itabapoana, RJ		5	-/-
	Domingos Martins, ES		2	
	Pancas, ES		1	
	Santa Maria de Jetibá, ES		2	
Evandromyia				19.2 (15)
Evandromyia carmelinoi	Cáceres, MT		2	-/-
*Evandromyia edwardsi**				19.04 (5.14)
Evandromyia edwardsi PS1	Pancas, ES	1	3	-/-
Evandromyia edwardsi PS2	Pancas, ES		1	-/-
	Santa Maria de Jetibá, ES		1	
Evandromyia edwardsi PS3	Pancas, ES	1	2	0.3 (0.12)
	Santa Maria de Jetibá, ES		1	
	Santa Teresa, ES		1	
Evandromyia lenti	Pancas, ES	1		-/-
Evandromyia spp.	Alfredo Chaves, ES		1	-/-
	Pancas, ES		8	
	Santa Leopoldina, ES		2	
Evandromyia termitophila	Cáceres, MT		2	-/-
Evandromyia tupynambai	Pancas, ES	1		-/-
Expapillata				0.61 (0.25)

Expapilatta firmatoi	Pancas, ES	3	3	0.61 (0.25)
Lutzomyia				15.30 (9.42)
Lutzomyia alencari	Pancas, ES	3		0.46 (0.31)
Lutzomyia cruzi	Cáceres, MT	6		4.1 (2.61)
Lutzomyia dispar	Cáceres, MT	2		-/-
Lutzomyia longipalpis	Pancas, ES<br>Cáceres, MT	10<br>6		5.07 (2.33)
Lutzomyia renei	Lagoa Santa, MG	10		0.46 (0.21)
Lutzomyia sp.	Cáceres, MT		1	-/-
Micropygomyia				16.26 (13.53)
Micropygomyia capixaba	Pancas, ES	4		-/-
Micropygomyia echinatopharynx	Cáceres, MT		1	-/-
Micropygomyia ferreirana	Bom Jesus do Itabapoana, RJ<br>Domingos Martins, ES<br>Pancas, ES<br>Santa Maria de Jetibá, ES	 2<br>19<br>3	1	3.13 (1.36)
Micropygomyia peresi	Cáceres, MT	1		-/-
Micropygomyia quinquefer	Pancas, ES	22	3	0.46 (0.15)
Micropygomyia schreiberi	Mantenópolis, ES<br>Marilândia, ES<br>Pancas, ES	 <br>20	1<br>1<br>1	0.30 (0.15)
Migonemyia				1.54 (0.47)
Migonemyia migonei	Itaguaçu, ES<br>Pancas, ES<br>Santa Maria de Jetibá, ES	1<br>18<br>1	3<br>5	1.54 (0.47)
Nyssomyia				7.08 (3.56)
Nyssomyia intermedia	Pancas, ES<br>Santa Leopoldina, ES	25<br>11	2<br>10	1.86 (0.79)
Nyssomyia whitmani	Cáceres, MT<br>Iúna, ES	7<br>1	1	2.97 (1.64)
Nyssomyia yuilli yuilli	Wenceslau Guimarães, BA	1		-/-
Pintomyia				
Pintomyia bianchigalatiae	Alto Rio Novo, ES<br>Domingos Martins, ES<br>Mantenópolis, ES		1<br>2<br>1	2.01 (1.39)
Pintomyia fischeri	Alto Rio Novo, ES		1	3.77 (1.05)

	Domingos Martins, ES	4	7	
	Itaguaçu, ES	1	1	
	Mantenópolis, ES	1		
	Marilândia, ES		1	
	Pancas, ES	12		
	Santa Leopoldina, ES	2	3	
	Santa Teresa, ES		3	
Pintomyia misionensis	Alto Rio Novo, ES	1		2.97 (0.87)
	Domingos Martins, ES		3	
	Ibitirama, ES		6	
	Itaguaçu, ES		10	
	Iúna, ES		5	
*Pintomyia monticola**				8.8 (4.26)
Pintomyia monticola PS1	Alfredo Chaves, ES		2	2.97 (1.31)
	Domingos Martins, ES		10	
	Santa Teresa, ES		8	
Pintomyia monticola PS2	Alto Rio Novo, ES		8	0.46 (0.12)
	Iúna, ES		2	
	João Neiva, ES		2	
	Mantenópolis, ES		8	
	Marilândia, ES		8	
	Santa Teresa, ES	1		
Pressatia				0.46 (0.15)
Pressatia choti	Pancas, ES	17		0.46 (0.18)
Pressatia spp.	Pancas, ES		8	-/-
Psathyromyia				17.01 (11.51)
*Psathyromyia bigeniculata**				4.23 (1.71)
Psathyromyia bigeniculata PS1	Santa Leopoldina, ES	1	5	0.3 (0.1)
Psathyromyia bigeniculata PS2	Cáceres, MT	1	1	-/-
Psathyromyia limai	Alfredo Chaves, ES		4	0.92 (0.31)
	Domingos Martins, ES	2		
	Itaguaçu, ES	2	5	
	João Neiva, ES		2	
	Marilândia, ES		2	
	Santa Maria de Jetibá, ES		4	
Psathyromyia lutziana	Pancas, ES	1	1	-/-
Psathyromyia pascalei	Alfredo Chaves, ES	3	1	0.76 (0.2)
	Alto Rio Novo, ES	1		
	Baixo Guandu, ES	1	1	
	Bom Jesus do Itabapoana, RJ	1		
	Domingos Martins, ES	1	3	
	Pancas, ES	3		
	Santa Maria de Jetibá, ES	1		
Psathyromyia pelloni	João Neiva, ES		1	-/-

Psychodopygus				14.78 (11.48)
Psychodopygus ayrozai	Domingos Martins, ES	6		2.17 (1.35)
	Wenceslau Guimarães, BA	1		
Psychodopygus davisi	Santa Leopoldina, ES	5	2	0.61 (0.39)
Psychodopygus hirsutus	Domingos Martins, ES	6	8	0.61 (0.12)
	João Neiva, ES		5	
	Pancas, ES	2	1	
	Santa Leopoldina, ES	1	2	
*Psychodopygus matosi**				4.41 (1.22)
Psychodopygus matosi PS1	Afonso Cláudio, ES	4	4	2.33 (1.1)
	Alfredo Chaves, ES	2	1	
	Marilândia, ES		1	
	Santa Maria de Jetibá, ES		2	
	Santa Teresa, ES	11	14	
Psychodopygus matosi PS2	Afonso Cláudio, ES	1		-/-
Sciopemyia				17.06 (13.77)
*Sciopemyia microps**				11.98 (8.03)
Sciopemyia microps PS1	Iúna, ES		1	-/-
Sciopemyia microps PS2	Pancas, ES	2		-/-
Sciopemyia sordellii	Cáceres, MT		1	-/-
Sciopemyia spp.	Pancas, ES		3	-/-
Trichophoromyia				1.39 (0.5)
Trichophoromyia viannamartinsi	Wenceslau Guimarães, BA	14	2	1.39 (0.5)
