# Supplementary material for: DNA Barcoding of Neotropical Sand Flies (Diptera, Psychodidae, Phlebotominae): Species Identification and Discovery within Brazil
Source: PLoS One. 2015 Oct 27;10(10):e0140636. doi: 10.1371/journal.pone.0140636 (PMC4624639; doi:10.1371/journal.pone.0140636)
Supplement: S2 Table — (PDF) [file pone.0140636.s006.pdf]

S2 Table. Phlebotominae taxa sampled, museum specimen number, BOLD and GenBank accession numbers, and geographical data. (BA = Bahia state; ES = Espírito Santo state; MG = Minas Gerais state; MT = Mato Grosso state; RJ = Rio de Janeiro state)

Identification	BIN	Sample ID	Museum ID	Process ID	GenBank	Municipality, State	Latitude	Longitude	Elevation
Bichromomyia flaviscutellata	BOLD:ACM6850	LBMI#0442	90526	AFBR541-14	KP112487	Santa Teresa, ES	19° 54’ 30” S	40° 39’ 25’’ W	754 m
Bichromomyia flaviscutellata	BOLD:ACM6850	LBMI#0441	90525	AFBR540-14	KP112488	Santa Teresa, ES	19° 54’ 30” S	40° 39’ 25’’ W	754 m
Brumptomyia cunhai	BOLD:ACK2711	LBMI#0261	90408	AFBR384-14	KP112489	Pancas, ES	19° 13’ 44’’ S	40° 45’ 31’’ W	133 m
Brumptomyia cunhai	BOLD:ACK2711	LBMI#0267	90416	AFBR390-14	KP112490	Pancas, ES	19° 13’ 44’’ S	40° 45’ 31’’ W	133 m
Brumptomyia cunhai	BOLD:ACK2711	LBMI#0004	90201	AFBR003-13	KP112491	Pancas, ES	19° 13’ 44’’ S	40° 45’ 31’’ W	133 m
Brumptomyia cunhai	BOLD:ACK2711	LBMI#0230	90386	AFBR358-14	KP112492	Pancas, ES	19° 13’ 44’’ S	40° 45’ 31’’ W	133 m
Brumptomyia cunhai	BOLD:ACK2711	LBMI#0231	90387	AFBR359-14	KP112493	Pancas, ES	19° 13’ 44’’ S	40° 45’ 31’’ W	133 m
Brumptomyia cunhai	BOLD:ACK2711	LBMI#0232	90388	AFBR360-14	KP112494	Pancas, ES	19° 13’ 44’’ S	40° 45’ 31’’ W	133 m
Brumptomyia cunhai	BOLD:ACK2711	LBMI#0234	90390	AFBR361-14	KP112495	Pancas, ES	19° 13’ 44’’ S	40° 45’ 31’’ W	133 m
Brumptomyia cunhai	BOLD:ACK2711	LBMI#0200	90354	AFBR335-14	KP112496	Pancas, ES	19° 13’ 44’’ S	40° 45’ 31’’ W	133 m
Brumptomyia cunhai	BOLD:ACK2711	LBMI#0268	90417	AFBR391-14	KP112497	Pancas, ES	19° 13’ 44’’ S	40° 45’ 31’’ W	133 m
Brumptomyia cunhai	BOLD:ACK2711	LBMI#0197	90353	AFBR332-14	KP112498	Pancas, ES	19° 13’ 44’’ S	40° 45’ 31’’ W	133 m
Brumptomyia cunhai	BOLD:ACK2711	LBMI#0239	90391	AFBR365-14	KP112499	Pancas, ES	19° 13’ 44’’ S	40° 45’ 31’’ W	133 m
Brumptomyia cunhai	BOLD:ACK2711	LBMI#0240	90392	AFBR366-14	KP112500	Pancas, ES	19° 13’ 44’’ S	40° 45’ 31’’ W	133 m
Brumptomyia cunhai	BOLD:ACK2711	LBMI#0269	90418	AFBR392-14	KP112501	Pancas, ES	19° 13’ 44’’ S	40° 45’ 31’’ W	133 m
Brumptomyia cunhai	BOLD:ACK2711	LBMI#0270	90419	AFBR393-14	KP112502	Pancas, ES	19° 13’ 44’’ S	40° 45’ 31’’ W	133 m
Brumptomyia cunhai	BOLD:ACK2711	LBMI#0283	90372	AFBR406-14	KP112503	Pancas, ES	19° 13’ 44’’ S	40° 45’ 31’’ W	133 m
Brumptomyia cunhai	BOLD:ACK2711	LBMI#0247	90394	AFBR372-14	KP112504	Pancas, ES	19° 13’ 44’’ S	40° 45’ 31’’ W	133 m
Brumptomyia cunhai	BOLD:ACK2711	LBMI#0064	90202	AFBR054-13	KP112505	Pancas, ES	19° 13’ 44’’ S	40° 45’ 31’’ W	133 m
Brumptomyia nitzulescui	BOLD:ACK6733	LBMI#0631	90726	AFBR702-14	KP112506	Domingos Martins, ES	20° 24’ 01” S	40° 45’ 11’’ W	673 m
Brumptomyia nitzulescui	BOLD:ACK6733	LBMI#0328	90473	AFBR447-14	KP112507	Santa Maria de Jetibá, ES	19° 58’ 54” S	40° 48’ 46” W	844 m
Brumptomyia nitzulescui	BOLD:ACK6733	LBMI#0549	90637	AFBR638-14	KP112508	Santa Maria de Jetibá, ES	19° 58’ 54” S	40° 48’ 46” W	844 m
Brumptomyia nitzulescui	BOLD:ACK6733	LBMI#0329	90474	AFBR448-14	KP112509	Santa Maria de Jetibá, ES	19° 58’ 54” S	40° 48’ 46” W	844 m
Brumptomyia nitzulescui	BOLD:ACK6733	LBMI#0460	90571	AFBR559-14	KP112510	Alto Rio Novo, ES	18° 58’ 35” S	41° 00’ 43’’ W	762 m
Brumptomyia nitzulescui	BOLD:ACK6733	LBMI#0332	90475	AFBR451-14	KP112511	Santa Maria de Jetibá, ES	19° 58’ 54” S	40° 48’ 46” W	844 m
Brumptomyia nitzulescui	BOLD:ACK6733	LBMI#0333	90476	AFBR452-14	KP112512	Santa Maria de Jetibá, ES	19° 58’ 54” S	40° 48’ 46” W	844 m
Brumptomyia nitzulescui	BOLD:ACK6733	LBMI#0334	90477	AFBR453-14	KP112513	Santa Maria de Jetibá, ES	19° 58’ 54” S	40° 48’ 46” W	844 m
Brumptomyia nitzulescui	BOLD:ACK6733	LBMI#0335	90478	AFBR454-14	KP112514	Santa Maria de Jetibá, ES	19° 58’ 54” S	40° 48’ 46” W	844 m
Brumptomyia nitzulescui	BOLD:ACK6733	LBMI#0336	90479	AFBR455-14	KP112515	Santa Maria de Jetibá, ES	19° 58’ 54” S	40° 48’ 46” W	844 m
Brumptomyia nitzulescui	BOLD:ACK6733	LBMI#0337	90480	AFBR456-14	KP112516	Santa Maria de Jetibá, ES	19° 58’ 54” S	40° 48’ 46” W	844 m

Brumptomyia nitzulescui	BOLD:ACK6733	LBMI#0338	90481	AFBR457-14	KP112517	Santa Maria de Jetibá, ES	19° 58’ 54” S	40° 48’ 46” W	844 m
Brumptomyia nitzulescui	BOLD:ACK6733	LBMI#0339	90482	AFBR458-14	KP112518	Santa Maria de Jetibá, ES	19° 58’ 54” S	40° 48’ 46” W	844 m
Brumptomyia nitzulescui	BOLD:ACK6733	LBMI#0636	90541	AFBR705-14	KP112519	Domingos Martins, ES	20° 24’ 01” S	40° 45’ 11’’ W	673 m
Brumptomyia nitzulescui	BOLD:ACK6733	LBMI#0633	90728	AFBR704-14	KP112520	Domingos Martins, ES	20° 24’ 01” S	40° 45’ 11’’ W	673 m
Brumptomyia nitzulescui	BOLD:ACK6733	LBMI#0632	90727	AFBR703-14	KP112521	Domingos Martins, ES	20° 24’ 01” S	40° 45’ 11’’ W	673 m
Brumptomyia ortizi	BOLD:ACM5394	LBMI#0391	90737	AFBR494-14	KP112522	Bom Jesus do Itabapoana, RJ	21° 03’ 25’’ S	41° 47’ 31’’ W	500 m
Brumptomyia ortizi	BOLD:ACM5394	LBMI#0394	90740	AFBR497-14	KP112523	Bom Jesus do Itabapoana, RJ	21° 03’ 25’’ S	41° 47’ 31’’ W	500 m
Brumptomyia sp.	BOLD:ACK6733	LBMI#0330	90485	AFBR449-14	KP112524	Santa Maria de Jetibá, ES	19° 58’ 54” S	40° 48’ 46” W	844 m
Brumptomyia sp.	BOLD:ACK6733	LBMI#0639	90544	AFBR707-14	KP112525	Domingos Martins, ES	20° 24’ 01” S	40° 45’ 11’’ W	673 m
Brumptomyia sp.	BOLD:ACK6733	LBMI#0331	90486	AFBR450-14	KP112527	Santa Maria de Jetibá, ES	19° 58’ 54” S	40° 48’ 46” W	844 m
Brumptomyia sp.	BOLD:ACK6733	LBMI#0638	90543	AFBR706-14	KP112528	Domingos Martins, ES	20° 24’ 01” S	40° 45’ 11’’ W	673 m
Brumptomyia sp.	BOLD:ACK6733	LBMI#0201	90355	AFBR336-14	KP112533	Pancas, ES	19° 13’ 44’’ S	40° 45’ 31’’ W	133 m
Brumptomyia sp.	BOLD:ACM5394	LBMI#0392	90738	AFBR495-14	KP112526	Bom Jesus do Itabapoana, RJ	21° 03’ 25’’ S	41° 47’ 31’’ W	500 m
Brumptomyia sp.	BOLD:ACM5394	LBMI#0389	90735	AFBR492-14	KP112529	Bom Jesus do Itabapoana, RJ	21° 03’ 25’’ S	41° 47’ 31’’ W	500 m
Brumptomyia sp.	BOLD:ACM5394	LBMI#0390	90736	AFBR493-14	KP112530	Bom Jesus do Itabapoana, RJ	21° 03’ 25’’ S	41° 47’ 31’’ W	500 m
Brumptomyia sp.	BOLD:ACM5394	LBMI#0393	90739	AFBR496-14	KP112531	Bom Jesus do Itabapoana, RJ	21° 03’ 25’’ S	41° 47’ 31’’ W	500 m
Brumptomyia sp.	BOLD:ACM5394	LBMI#0388	90734	AFBR491-14	KP112532	Bom Jesus do Itabapoana, RJ	21° 03’ 25’’ S	41° 47’ 31’’ W	500 m
Evandromyia carmelinoi	BOLD:ACK2880	LBMI#0398	90766	AFBR498-14	KP112534	Cáceres, MT	16° 24’ 08’’ S	57° 29’ 55’’ W	286 m
Evandromyia carmelinoi	BOLD:ACK2880	LBMI#0400	90768	AFBR499-14	KP112535	Cáceres, MT	16° 24’ 08’’ S	57° 29’ 55’’ W	286 m
Evandromyia edwardsi	BOLD:ACK5589	LBMI#0340	90487	AFBR459-14	KP112536	Santa Maria de Jetibá, ES	19° 58’ 54” S	40° 48’ 46” W	844 m
Evandromyia edwardsi	BOLD:ACK5589	LBMI#0253	90407	AFBR376-14	KP112537	Pancas, ES	19° 13’ 44’’ S	40° 45’ 31’’ W	133 m
Evandromyia edwardsi	BOLD:ACK5589	LBMI#0347	90494	AFBR466-14	KP112540	Santa Teresa, ES	19° 54’ 30” S	40° 39’ 25’’ W	754 m
Evandromyia edwardsi	BOLD:ACK5589	LBMI#0164	90286	AFBR308-14	KP112543	Pancas, ES	19° 13’ 44’’ S	40° 45’ 31’’ W	133 m
Evandromyia edwardsi	BOLD:ACK5589	LBMI#0199	90356	AFBR334-14	KP112544	Pancas, ES	19° 13’ 44’’ S	40° 45’ 31’’ W	133 m
Evandromyia edwardsi	BOLD:ACK6779	LBMI#0244	90396	AFBR369-14	KP112539	Pancas, ES	19° 13’ 44’’ S	40° 45’ 31’’ W	133 m
Evandromyia edwardsi	BOLD:ACK6779	LBMI#0115	90203	AFBR266-14	KP112542	Pancas, ES	19° 13’ 44’’ S	40° 45’ 31’’ W	133 m
Evandromyia edwardsi	BOLD:ACK6779	LBMI#0262	90420	AFBR385-14	KP112546	Pancas, ES	19° 13’ 44’’ S	40° 45’ 31’’ W	133 m
Evandromyia edwardsi	BOLD:ACK6779	LBMI#0260	90409	AFBR383-14	KP112547	Pancas, ES	19° 13’ 44’’ S	40° 45’ 31’’ W	133 m
Evandromyia edwardsi	BOLD:ACK6780	LBMI#0252	90406	AFBR375-14	KP112538	Pancas, ES	19° 13’ 44’’ S	40° 45’ 31’’ W	133 m
Evandromyia edwardsi	BOLD:ACK6780	LBMI#0341	90488	AFBR460-14	KP112541	Santa Maria de Jetibá, ES	19° 58’ 54” S	40° 48’ 46” W	844 m
Evandromyia lenti	BOLD:ACK2880	LBMI#0026	90189	AFBR024-13	KP112548	Pancas, ES	19° 13’ 44’’ S	40° 45’ 31’’ W	133 m
Evandromyia sp.	BOLD:ACK6874	LBMI#0342	90173	AFBR461-14	KP112549	Pancas, ES	19° 13’ 44’’ S	40° 45’ 31’’ W	133 m
Evandromyia sp.	BOLD:ACK6874	LBMI#0280	90373	AFBR403-14	KP112552	Pancas, ES	19° 13’ 44’’ S	40° 45’ 31’’ W	133 m

Evandromyia sp.	BOLD:ACK6874	LBMI#0245	90398	AFBR370-14	KP112553	Pancas, ES	19° 13’ 44’’ S	40° 45’ 31’’ W	133 m
Evandromyia sp.	BOLD:ACK6874	LBMI#0140	90288	AFBR290-14	KP112554	Pancas, ES	19° 13’ 44’’ S	40° 45’ 31’’ W	133 m
Evandromyia sp.	BOLD:ACK6874	LBMI#0193	90357	AFBR329-14	KP112556	Pancas, ES	19° 13’ 44’’ S	40° 45’ 31’’ W	133 m
Evandromyia sp.	BOLD:ACK6874	LBMI#0237	90397	AFBR363-14	KP112557	Pancas, ES	19° 13’ 44’’ S	40° 45’ 31’’ W	133 m
Evandromyia sp.	BOLD:ACK6874	LBMI#0263	90421	AFBR386-14	KP112558	Pancas, ES	19° 13’ 44’’ S	40° 45’ 31’’ W	133 m
Evandromyia sp.	BOLD:ACK6874	LBMI#0118	90287	AFBR269-14	KP112559	Pancas, ES	19° 13’ 44’’ S	40° 45’ 31’’ W	133 m
Evandromyia sp.	BOLD:ACK6875	LBMI#0493	90581	AFBR589-14	KP112550	Santa Leopoldina, ES	20° 08’ 16” S	40° 30’ 57 W	51 m
Evandromyia sp.	BOLD:ACK6875	LBMI#0494	90582	AFBR590-14	KP112551	Santa Leopoldina, ES	20° 08’ 16” S	40° 30’ 57 W	51 m
Evandromyia sp.	BOLD:ACK6875	LBMI#0303	90456	AFBR424-14	KP112555	Alfredo Chaves, ES	20° 29’ 25” S	40° 57’ 28’’ W	1069 m
Evandromyia termitophila	BOLD:ACM6762	LBMI#0401	90769	AFBR500-14	KP112560	Cáceres, MT	16° 24’ 08’’ S	57° 29’ 55’’ W	286 m
Evandromyia termitophila	BOLD:ACM6762	LBMI#0396	90.764	AFBR752-14	KP112561	Cáceres, MT	16° 24’ 08’’ S	57° 29’ 55’’ W	286 m
Evandromyia tupynambai	BOLD:ACK6874	LBMI#0277	90374	AFBR400-14	KP112562	Pancas, ES	19° 13’ 44’’ S	40° 45’ 31’’ W	133 m
Expapillata firmatoi	BOLD:ACK2994	LBMI#0159	90292	AFBR306-14	KP112563	Pancas, ES	19° 13’ 44’’ S	40° 45’ 31’’ W	133 m
Expapillata firmatoi	BOLD:ACK2994	LBMI#0119	90289	AFBR270-14	KP112564	Pancas, ES	19° 13’ 44’’ S	40° 45’ 31’’ W	133 m
Expapillata firmatoi	BOLD:ACK2994	LBMI#0154	90291	AFBR302-14	KP112565	Pancas, ES	19° 13’ 44’’ S	40° 45’ 31’’ W	133 m
Expapillata firmatoi	BOLD:ACK2994	LBMI#0139	90290	AFBR289-14	KP112566	Pancas, ES	19° 13’ 44’’ S	40° 45’ 31’’ W	133 m
Expapillata firmatoi	BOLD:ACK2994	LBMI#0183	90318	AFBR320-14	KP112567	Pancas, ES	19° 13’ 44’’ S	40° 45’ 31’’ W	133 m
Expapillata firmatoi	BOLD:ACK2994	LBMI#0167	90317	AFBR310-14	KP112568	Pancas, ES	19° 13’ 44’’ S	40° 45’ 31’’ W	133 m
Lutzomyia alencari	BOLD:ACD9711	LBMI#0343	90174	AFBR462-14	KP112569	Pancas, ES	19° 13’ 44’’ S	40° 45’ 31’’ W	133 m
Lutzomyia alencari	BOLD:ACD9711	LBMI#0430	90505	AFBR529-14	KP112570	Pancas, ES	19° 13’ 44’’ S	40° 45’ 31’’ W	133 m
Lutzomyia alencari	BOLD:ACD9711	LBMI#0344	90175	AFBR463-14	KP112571	Pancas, ES	19° 13’ 44’’ S	40° 45’ 31’’ W	133 m
Lutzomyia cruzi	BOLD:AAY5016	LBMI#0420	90788	AFBR519-14	KP112573	Cáceres, MT	16° 24’ 08’’ S	57° 29’ 55’’ W	286 m
Lutzomyia cruzi	BOLD:AAY5016	LBMI#0416	90784	AFBR515-14	KP112575	Cáceres, MT	16° 24’ 08’’ S	57° 29’ 55’’ W	286 m
Lutzomyia cruzi	BOLD:ACM6900	LBMI#0413	90781	AFBR512-14	KP112577	Cáceres, MT	16° 24’ 08’’ S	57° 29’ 55’’ W	286 m
Lutzomyia cruzi	BOLD:ACM6901	LBMI#0423	90791	AFBR522-14	KP112572	Cáceres, MT	16° 24’ 08’’ S	57° 29’ 55’’ W	286 m
Lutzomyia cruzi	BOLD:ACM6901	LBMI#0406	90774	AFBR505-14	KP112574	Cáceres, MT	16° 24’ 08’’ S	57° 29’ 55’’ W	286 m
Lutzomyia cruzi	BOLD:ACM6901	LBMI#0411	90779	AFBR510-14	KP112576	Cáceres, MT	16° 24’ 08’’ S	57° 29’ 55’’ W	286 m
Lutzomyia dispar	BOLD:ACM5192	LBMI#0364	90761	AFBR477-14	KP112578	Cáceres, MT	16° 24’ 08’’ S	57° 29’ 55’’ W	286 m
Lutzomyia dispar	BOLD:ACM5192	LBMI#0365	90762	AFBR478-14	KP112579	Cáceres, MT	16° 24’ 08’’ S	57° 29’ 55’’ W	286 m
Lutzomyia longipalpis	BOLD:AAY5016	LBMI#0410	90778	AFBR509-14	KP112586	Cáceres, MT	16° 24’ 08’’ S	57° 29’ 55’’ W	286 m
Lutzomyia longipalpis	BOLD:AAY5016	LBMI#0422	90790	AFBR521-14	KP112595	Cáceres, MT	16° 24’ 08’’ S	57° 29’ 55’’ W	286 m
Lutzomyia longipalpis	BOLD:ACD9711	LBMI#0044	90183	AFBR038-13	KP112580	Pancas, ES	19° 13’ 44’’ S	40° 45’ 31’’ W	133 m
Lutzomyia longipalpis	BOLD:ACD9711	LBMI#0049	90188	AFBR042-13	KP112582	Pancas, ES	19° 13’ 44’’ S	40° 45’ 31’’ W	133 m

Lutzomyia longipalpis	BOLD:ACD9711	LBMI#0047	90186	AFBR041-13	KP112583	Pancas, ES	19° 13’ 44’’ S	40° 45’ 31’’ W	133 m
Lutzomyia longipalpis	BOLD:ACD9711	LBMI#0046	90185	AFBR040-13	KP112584	Pancas, ES	19° 13’ 44’’ S	40° 45’ 31’’ W	133 m
Lutzomyia longipalpis	BOLD:ACD9711	LBMI#0045	90184	AFBR039-13	KP112585	Pancas, ES	19° 13’ 44’’ S	40° 45’ 31’’ W	133 m
Lutzomyia longipalpis	BOLD:ACD9711	LBMI#0409	90777	AFBR508-14	KP112587	Cáceres, MT	16° 24’ 08’’ S	57° 29’ 55’’ W	286 m
Lutzomyia longipalpis	BOLD:ACD9711	LBMI#0403	90771	AFBR502-14	KP112589	Cáceres, MT	16° 24’ 08’’ S	57° 29’ 55’’ W	286 m
Lutzomyia longipalpis	BOLD:ACD9711	LBMI#0043	90182	AFBR037-13	KP112590	Pancas, ES	19° 13’ 44’’ S	40° 45’ 31’’ W	133 m
Lutzomyia longipalpis	BOLD:ACD9711	LBMI#0042	90181	AFBR036-13	KP112591	Pancas, ES	19° 13’ 44’’ S	40° 45’ 31’’ W	133 m
Lutzomyia longipalpis	BOLD:ACD9711	LBMI#0041	90180	AFBR035-13	KP112592	Pancas, ES	19° 13’ 44’’ S	40° 45’ 31’’ W	133 m
Lutzomyia longipalpis	BOLD:ACD9711	LBMI#0039	90178	AFBR034-13	KP112593	Pancas, ES	19° 13’ 44’’ S	40° 45’ 31’’ W	133 m
Lutzomyia longipalpis	BOLD:ACD9711	LBMI#0038	90177	AFBR033-13	KP112594	Pancas, ES	19° 13’ 44’’ S	40° 45’ 31’’ W	133 m
Lutzomyia longipalpis	BOLD:ACM6900	LBMI#0408	90776	AFBR507-14	KP112588	Cáceres, MT	16° 24’ 08’’ S	57° 29’ 55’’ W	286 m
Lutzomyia longipalpis	BOLD:ACM6901	LBMI#0418	90786	AFBR517-14	KP112581	Cáceres, MT	16° 24’ 08’’ S	57° 29’ 55’’ W	286 m
Lutzomyia renei	BOLD:ACM7046	LBMI#0490	90802	AFBR587-14	KP112596	Lagoa Santa, MG	10° 45’ 48’’ S	48° 06’ 32’’ W	740 m
Lutzomyia renei	BOLD:ACM7046	LBMI#0486	90798	AFBR583-14	KP112597	Lagoa Santa, MG	10° 45’ 48’’ S	48° 06’ 32’’ W	740 m
Lutzomyia renei	BOLD:ACM7046	LBMI#0484	90796	AFBR581-14	KP112598	Lagoa Santa, MG	10° 45’ 48’’ S	48° 06’ 32’’ W	740 m
Lutzomyia renei	BOLD:ACM7046	LBMI#0487	90799	AFBR584-14	KP112599	Lagoa Santa, MG	10° 45’ 48’’ S	48° 06’ 32’’ W	740 m
Lutzomyia renei	BOLD:ACM7046	LBMI#0483	90795	AFBR580-14	KP112600	Lagoa Santa, MG	10° 45’ 48’’ S	48° 06’ 32’’ W	740 m
Lutzomyia renei	BOLD:ACM7046	LBMI#0485	90797	AFBR582-14	KP112601	Lagoa Santa, MG	10° 45’ 48’’ S	48° 06’ 32’’ W	740 m
Lutzomyia renei	BOLD:ACM7046	LBMI#0489	90801	AFBR586-14	KP112602	Lagoa Santa, MG	10° 45’ 48’’ S	48° 06’ 32’’ W	740 m
Lutzomyia renei	BOLD:ACM7046	LBMI#0488	90800	AFBR585-14	KP112603	Lagoa Santa, MG	10° 45’ 48’’ S	48° 06’ 32’’ W	740 m
Lutzomyia renei	BOLD:ACM7046	LBMI#0481	90793	AFBR578-14	KP112604	Lagoa Santa, MG	10° 45’ 48’’ S	48° 06’ 32’’ W	740 m
Lutzomyia renei	BOLD:ACM7046	LBMI#0482	90794	AFBR579-14	KP112605	Lagoa Santa, MG	10° 45’ 48’’ S	48° 06’ 32’’ W	740 m
Lutzomyia sp.	BOLD:ACM6901	LBMI#0414	90782	AFBR513-14	KP112606	Cáceres, MT	16° 24’ 08’’ S	57° 29’ 55’’ W	286 m
Micropygomyia capixaba	BOLD:ACK6942	LBMI#0282	90378	AFBR405-14	KP112607	Pancas, ES	19° 13’ 44’’ S	40° 45’ 31’’ W	133 m
Micropygomyia capixaba	BOLD:ACK6942	LBMI#0276	90376	AFBR399-14	KP112608	Pancas, ES	19° 13’ 44’’ S	40° 45’ 31’’ W	133 m
Micropygomyia capixaba	BOLD:ACK6942	LBMI#0278	90377	AFBR401-14	KP112609	Pancas, ES	19° 13’ 44’’ S	40° 45’ 31’’ W	133 m
Micropygomyia capixaba	BOLD:ACK6942	LBMI#0275	90375	AFBR398-14	KP112610	Pancas, ES	19° 13’ 44’’ S	40° 45’ 31’’ W	133 m
Micropygomyia echinatopharynx	BOLD:ACM7137	LBMI#0395	90.763	AFBR751-14	KP112611	Cáceres, MT	16° 24’ 08’’ S	57° 29’ 55’’ W	286 m
Micropygomyia ferreirana	BOLD:ACK2238	LBMI#0603	90691	AFBR678-14	KP112612	Domingos Martins, ES	20° 24’ 01” S	40° 45’ 11” W	673 m
Micropygomyia ferreirana	BOLD:ACK2238	LBMI#0286	90382	AFBR409-14	KP112613	Pancas, ES	19° 13’ 44’’ S	40° 45’ 31’’ W	133 m
Micropygomyia ferreirana	BOLD:ACK2238	LBMI#0285	90381	AFBR408-14	KP112614	Pancas, ES	19° 13’ 44’’ S	40° 45’ 31’’ W	133 m
Micropygomyia ferreirana	BOLD:ACK2238	LBMI#0321	90483	AFBR440-14	KP112615	Santa Maria de Jetibá, ES	19° 58’ 54” S	40° 48’ 46” W	844 m
Micropygomyia ferreirana	BOLD:ACK2238	LBMI#0322	90484	AFBR441-14	KP112616	Santa Maria de Jetibá, ES	19° 58’ 54” S	40° 48’ 46” W	844 m

Micropygomyia ferreirana	BOLD:ACK2238	LBMI#0284	90380	AFBR407-14	KP112617	Pancas, ES	19° 13’ 44’’ S	40° 45’ 31’’ W	133 m
Micropygomyia ferreirana	BOLD:ACK2238	LBMI#0585	90673	AFBR667-14	KP112618	Domingos Martins, ES	20° 24’ 01” S	40° 45’ 11’’ W	673 m
Micropygomyia ferreirana	BOLD:ACK2238	LBMI#0148	90293	AFBR297-14	KP112619	Pancas, ES	19° 13’ 44’’ S	40° 45’ 31’’ W	133 m
Micropygomyia ferreirana	BOLD:ACK2238	LBMI#0117	90294	AFBR268-14	KP112620	Pancas, ES	19° 13’ 44’’ S	40° 45’ 31’’ W	133 m
Micropygomyia ferreirana	BOLD:ACK2238	LBMI#0550	90638	AFBR639-14	KP112621	Santa Maria de Jetibá, ES	19° 58’ 54” S	40° 48’ 46” W	844 m
Micropygomyia ferreirana	BOLD:ACK2238	LBMI#0264	90422	AFBR387-14	KP112622	Pancas, ES	19° 13’ 44’’ S	40° 45’ 31’’ W	133 m
Micropygomyia ferreirana	BOLD:ACK2238	LBMI#0279	90379	AFBR402-14	KP112623	Pancas, ES	19° 13’ 44’’ S	40° 45’ 31’’ W	133 m
Micropygomyia ferreirana	BOLD:ACK2238	LBMI#0082	90207	AFBR069-13	KP112624	Pancas, ES	19° 13’ 44’’ S	40° 45’ 31’’ W	133 m
Micropygomyia ferreirana	BOLD:ACK2238	LBMI#0257	90410	AFBR380-14	KP112625	Pancas, ES	19° 13’ 44’’ S	40° 45’ 31’’ W	133 m
Micropygomyia ferreirana	BOLD:ACK2238	LBMI#0243	90402	AFBR368-14	KP112626	Pancas, ES	19° 13’ 44’’ S	40° 45’ 31’’ W	133 m
Micropygomyia ferreirana	BOLD:ACK2238	LBMI#0242	90401	AFBR367-14	KP112627	Pancas, ES	19° 13’ 44’’ S	40° 45’ 31’’ W	133 m
Micropygomyia ferreirana	BOLD:ACK2238	LBMI#0238	90400	AFBR364-14	KP112628	Pancas, ES	19° 13’ 44’’ S	40° 45’ 31’’ W	133 m
Micropygomyia ferreirana	BOLD:ACK2238	LBMI#0235	90399	AFBR362-14	KP112629	Pancas, ES	19° 13’ 44’’ S	40° 45’ 31’’ W	133 m
Micropygomyia ferreirana	BOLD:ACK2238	LBMI#0203	90358	AFBR338-14	KP112631	Pancas, ES	19° 13’ 44’’ S	40° 45’ 31’’ W	133 m
Micropygomyia ferreirana	BOLD:ACK2238	LBMI#0204	90359	AFBR339-14	KP112632	Pancas, ES	19° 13’ 44’’ S	40° 45’ 31’’ W	133 m
Micropygomyia ferreirana	BOLD:ACK2238	LBMI#0206	90361	AFBR340-14	KP112633	Pancas, ES	19° 13’ 44’’ S	40° 45’ 31’’ W	133 m
Micropygomyia ferreirana	BOLD:ACM4770	LBMI#0385	90731	AFBR488-14	KP112636	Bom Jesus do Itabapoana, RJ	21° 03’ 25’’ S	41° 47’ 31’’ W	500 m
Micropygomyia ferreirana	BOLD:ACM5781	LBMI#0246	90403	AFBR371-14	KP112630	Pancas, ES	19° 13’ 44’’ S	40° 45’ 31’’ W	133 m
Micropygomyia ferreirana	BOLD:ACM5781	LBMI#0207	90362	AFBR341-14	KP112634	Pancas, ES	19° 13’ 44’’ S	40° 45’ 31’’ W	133 m
Micropygomyia ferreirana	BOLD:ACM5781	LBMI#0208	90363	AFBR342-14	KP112635	Pancas, ES	19° 13’ 44’’ S	40° 45’ 31’’ W	133 m
Micropygomyia peresi	BOLD:ACM7136	LBMI#0397	90.765	AFBR753-14	KP112637	Cáceres, MT	16° 24’ 08’’ S	57° 29’ 55’’ W	286 m
Micropygomyia quinquefer	BOLD:ACK3053	LBMI#0190	90327	AFBR326-14	KP112638	Pancas, ES	19° 13’ 44’’ S	40° 45’ 31’’ W	133 m
Micropygomyia quinquefer	BOLD:ACK3053	LBMI#0191	90328	AFBR327-14	KP112639	Pancas, ES	19° 13’ 44’’ S	40° 45’ 31’’ W	133 m
Micropygomyia quinquefer	BOLD:ACK3053	LBMI#0192	90329	AFBR328-14	KP112640	Pancas, ES	19° 13’ 44’’ S	40° 45’ 31’’ W	133 m
Micropygomyia quinquefer	BOLD:ACK3053	LBMI#0281	90384	AFBR404-14	KP112641	Pancas, ES	19° 13’ 44’’ S	40° 45’ 31’’ W	133 m
Micropygomyia quinquefer	BOLD:ACK3053	LBMI#0194	90364	AFBR330-14	KP112642	Pancas, ES	19° 13’ 44’’ S	40° 45’ 31’’ W	133 m
Micropygomyia quinquefer	BOLD:ACK3053	LBMI#0195	90365	AFBR331-14	KP112643	Pancas, ES	19° 13’ 44’’ S	40° 45’ 31’’ W	133 m
Micropygomyia quinquefer	BOLD:ACK3053	LBMI#0018	90209	AFBR017-13	KP112644	Pancas, ES	19° 13’ 44’’ S	40° 45’ 31’’ W	133 m
Micropygomyia quinquefer	BOLD:ACK3053	LBMI#0150	90298	AFBR299-14	KP112645	Pancas, ES	19° 13’ 44’’ S	40° 45’ 31’’ W	133 m
Micropygomyia quinquefer	BOLD:ACK3053	LBMI#0149	90297	AFBR298-14	KP112646	Pancas, ES	19° 13’ 44’’ S	40° 45’ 31’’ W	133 m
Micropygomyia quinquefer	BOLD:ACK3053	LBMI#0073	90213	AFBR061-13	KP112647	Pancas, ES	19° 13’ 44’’ S	40° 45’ 31’’ W	133 m
Micropygomyia quinquefer	BOLD:ACK3053	LBMI#0147	90296	AFBR296-14	KP112648	Pancas, ES	19° 13’ 44’’ S	40° 45’ 31’’ W	133 m
Micropygomyia quinquefer	BOLD:ACK3053	LBMI#0168	90319	AFBR311-14	KP112649	Pancas, ES	19° 13’ 44’’ S	40° 45’ 31’’ W	133 m

Micropygomyia quinquefer	BOLD:ACK3053	LBMI#0227	90366	AFBR355-14	KP112650	Pancas, ES	19° 13’ 44’’ S	40° 45’ 31’’ W	133 m
Micropygomyia quinquefer	BOLD:ACK3053	LBMI#0171	90320	AFBR313-14	KP112651	Pancas, ES	19° 13’ 44’’ S	40° 45’ 31’’ W	133 m
Micropygomyia quinquefer	BOLD:ACK3053	LBMI#0072	90212	AFBR060-13	KP112652	Pancas, ES	19° 13’ 44’’ S	40° 45’ 31’’ W	133 m
Micropygomyia quinquefer	BOLD:ACK3053	LBMI#0116	90214	AFBR267-14	KP112653	Pancas, ES	19° 13’ 44’’ S	40° 45’ 31’’ W	133 m
Micropygomyia quinquefer	BOLD:ACK3053	LBMI#0155	90350	AFBR303-14	KP112654	Pancas, ES	19° 13’ 44’’ S	40° 45’ 31’’ W	133 m
Micropygomyia quinquefer	BOLD:ACK3053	LBMI#0180	90323	AFBR317-14	KP112655	Pancas, ES	19° 13’ 44’’ S	40° 45’ 31’’ W	133 m
Micropygomyia quinquefer	BOLD:ACK3053	LBMI#0274	90383	AFBR397-14	KP112656	Pancas, ES	19° 13’ 44’’ S	40° 45’ 31’’ W	133 m
Micropygomyia quinquefer	BOLD:ACK3053	LBMI#0182	90322	AFBR319-14	KP112657	Pancas, ES	19° 13’ 44’’ S	40° 45’ 31’’ W	133 m
Micropygomyia quinquefer	BOLD:ACK3053	LBMI#0071	90211	AFBR059-13	KP112658	Pancas, ES	19° 13’ 44’’ S	40° 45’ 31’’ W	133 m
Micropygomyia quinquefer	BOLD:ACK3053	LBMI#0016	90208	AFBR015-13	KP112659	Pancas, ES	19° 13’ 44’’ S	40° 45’ 31’’ W	133 m
Micropygomyia quinquefer	BOLD:ACK3053	LBMI#0185	90324	AFBR322-14	KP112660	Pancas, ES	19° 13’ 44’’ S	40° 45’ 31’’ W	133 m
Micropygomyia quinquefer	BOLD:ACK3053	LBMI#0187	90326	AFBR324-14	KP112661	Pancas, ES	19° 13’ 44’’ S	40° 45’ 31’’ W	133 m
Micropygomyia quinquefer	BOLD:ACK3053	LBMI#0186	90325	AFBR323-14	KP112662	Pancas, ES	19° 13’ 44’’ S	40° 45’ 31’’ W	133 m
Micropygomyia schreiberi	BOLD:ACK2625	LBMI#0144	90300	AFBR294-14	KP112663	Pancas, ES	19° 13’ 44’’ S	40° 45’ 31’’ W	133 m
Micropygomyia schreiberi	BOLD:ACK2625	LBMI#0021	90216	AFBR020-13	KP112664	Pancas, ES	19° 13’ 44’’ S	40° 45’ 31’’ W	133 m
Micropygomyia schreiberi	BOLD:ACK2625	LBMI#0022	90217	AFBR021-13	KP112665	Pancas, ES	19° 13’ 44’’ S	40° 45’ 31’’ W	133 m
Micropygomyia schreiberi	BOLD:ACK2625	LBMI#0023	90218	AFBR022-13	KP112666	Pancas, ES	19° 13’ 44’’ S	40° 45’ 31’’ W	133 m
Micropygomyia schreiberi	BOLD:ACK2625	LBMI#0024	90190	AFBR023-13	KP112667	Pancas, ES	19° 13’ 44’’ S	40° 45’ 31’’ W	133 m
Micropygomyia schreiberi	BOLD:ACK2625	LBMI#0056	90219	AFBR049-13	KP112668	Pancas, ES	19° 13’ 44’’ S	40° 45’ 31’’ W	133 m
Micropygomyia schreiberi	BOLD:ACK2625	LBMI#0060	90223	AFBR052-13	KP112669	Pancas, ES	19° 13’ 44’’ S	40° 45’ 31’’ W	133 m
Micropygomyia schreiberi	BOLD:ACK2625	LBMI#0081	90225	AFBR068-13	KP112670	Pancas, ES	19° 13’ 44’’ S	40° 45’ 31’’ W	133 m
Micropygomyia schreiberi	BOLD:ACK2625	LBMI#0108	90226	AFBR259-14	KP112671	Pancas, ES	19° 13’ 44’’ S	40° 45’ 31’’ W	133 m
Micropygomyia schreiberi	BOLD:ACK2625	LBMI#0109	90227	AFBR260-14	KP112672	Pancas, ES	19° 13’ 44’’ S	40° 45’ 31’’ W	133 m
Micropygomyia schreiberi	BOLD:ACK2625	LBMI#0110	90228	AFBR261-14	KP112673	Pancas, ES	19° 13’ 44’’ S	40° 45’ 31’’ W	133 m
Micropygomyia schreiberi	BOLD:ACK2625	LBMI#0131	90229	AFBR282-14	KP112674	Pancas, ES	19° 13’ 44’’ S	40° 45’ 31’’ W	133 m
Micropygomyia schreiberi	BOLD:ACK2625	LBMI#0132	90230	AFBR283-14	KP112675	Pancas, ES	19° 13’ 44’’ S	40° 45’ 31’’ W	133 m
Micropygomyia schreiberi	BOLD:ACK2625	LBMI#0133	90231	AFBR284-14	KP112676	Pancas, ES	19° 13’ 44’’ S	40° 45’ 31’’ W	133 m
Micropygomyia schreiberi	BOLD:ACK2625	LBMI#0134	90232	AFBR285-14	KP112677	Pancas, ES	19° 13’ 44’’ S	40° 45’ 31’’ W	133 m
Micropygomyia schreiberi	BOLD:ACK2625	LBMI#0142	90301	AFBR292-14	KP112678	Pancas, ES	19° 13’ 44’’ S	40° 45’ 31’’ W	133 m
Micropygomyia schreiberi	BOLD:ACK2625	LBMI#0143	90299	AFBR293-14	KP112679	Pancas, ES	19° 13’ 44’’ S	40° 45’ 31’’ W	133 m
Micropygomyia schreiberi	BOLD:ACK2625	LBMI#0146	90302	AFBR295-14	KP112680	Pancas, ES	19° 13’ 44’’ S	40° 45’ 31’’ W	133 m
Micropygomyia schreiberi	BOLD:ACK2625	LBMI#0156	90303	AFBR304-14	KP112681	Pancas, ES	19° 13’ 44’’ S	40° 45’ 31’’ W	133 m
Micropygomyia schreiberi	BOLD:ACK2625	LBMI#0160	90304	AFBR307-14	KP112682	Pancas, ES	19° 13’ 44’’ S	40° 45’ 31’’ W	133 m

Micropygomyia schreiberi	BOLD:ACK2625	LBMI#0459	90570	AFBR558-14	KP112683	Alto Rio Novo, ES	18° 58' 35" S	41° 00' 43'' W	762 m
Micropygomyia schreiberi	BOLD:ACK2625	LBMI#0477	90568	AFBR574-14	KP112684	Mantenópolis, ES	18° 51' 09" S	41° 03' 59" W	661 m
Micropygomyia schreiberi	BOLD:ACK2625	LBMI#0002	90215	AFBR002-13	KP112685	Pancas, ES	19° 13' 44'' S	40° 45' 31'' W	133 m
Micropygomyia schreiberi	BOLD:ACK2625	LBMI#0538	90626	AFBR629-14	KP112686	Marilândia, ES	19° 19' 04" S	40° 31' 01" W	581 m
Migonemyia migonei	BOLD:ACD7233	LBMI#0011	90233	AFBR010-13	KP112687	Pancas, ES	19° 13' 44'' S	40° 45' 31'' W	133 m
Migonemyia migonei	BOLD:ACD7233	LBMI#0566	90654	AFBR652-14	KP112688	Itaguaçu, ES	19° 44' 13" S	40° 58' 09" W	871 m
Migonemyia migonei	BOLD:ACD7233	LBMI#0567	90655	AFBR653-14	KP112689	Itaguaçu, ES	19° 44' 13" S	40° 58' 09" W	871 m
Migonemyia migonei	BOLD:ACD7233	LBMI#0125	90245	AFBR276-14	KP112690	Pancas, ES	19° 13' 44'' S	40° 45' 31'' W	133 m
Migonemyia migonei	BOLD:ACD7233	LBMI#0013	90251	AFBR012-13	KP112691	Pancas, ES	19° 13' 44'' S	40° 45' 31'' W	133 m
Migonemyia migonei	BOLD:ACD7233	LBMI#0138	90309	AFBR288-14	KP112692	Pancas, ES	19° 13' 44'' S	40° 45' 31'' W	133 m
Migonemyia migonei	BOLD:ACD7233	LBMI#0137	90308	AFBR287-14	KP112693	Pancas, ES	19° 13' 44'' S	40° 45' 31'' W	133 m
Migonemyia migonei	BOLD:ACD7233	LBMI#0126	90246	AFBR277-14	KP112694	Pancas, ES	19° 13' 44'' S	40° 45' 31'' W	133 m
Migonemyia migonei	BOLD:ACD7233	LBMI#0127	90247	AFBR278-14	KP112695	Pancas, ES	19° 13' 44'' S	40° 45' 31'' W	133 m
Migonemyia migonei	BOLD:ACD7233	LBMI#0128	90248	AFBR279-14	KP112696	Pancas, ES	19° 13' 44'' S	40° 45' 31'' W	133 m
Migonemyia migonei	BOLD:ACD7233	LBMI#0323	90489	AFBR442-14	KP112697	Santa Maria de Jetibá, ES	19° 58' 54" S	40° 48' 46" W	844 m
Migonemyia migonei	BOLD:ACD7233	LBMI#0129	90249	AFBR280-14	KP112698	Pancas, ES	19° 13' 44'' S	40° 45' 31'' W	133 m
Migonemyia migonei	BOLD:ACD7233	LBMI#0130	90250	AFBR281-14	KP112699	Pancas, ES	19° 13' 44'' S	40° 45' 31'' W	133 m
Migonemyia migonei	BOLD:ACD7233	LBMI#0568	90656	AFBR738-14	KP112700	Itaguaçu, ES	19° 44' 13" S	40° 58' 09" W	871 m
Migonemyia migonei	BOLD:ACD7233	LBMI#0135	90307	AFBR286-14	KP112701	Pancas, ES	19° 13' 44'' S	40° 45' 31'' W	133 m
Migonemyia migonei	BOLD:ACD7233	LBMI#0565	90653	AFBR651-14	KP112702	Itaguaçu, ES	19° 44' 13" S	40° 58' 09" W	871 m
Migonemyia migonei	BOLD:ACD7233	LBMI#0050	90234	AFBR043-13	KP112703	Pancas, ES	19° 13' 44'' S	40° 45' 31'' W	133 m
Migonemyia migonei	BOLD:ACD7233	LBMI#0052	90236	AFBR045-13	KP112704	Pancas, ES	19° 13' 44'' S	40° 45' 31'' W	133 m
Migonemyia migonei	BOLD:ACD7233	LBMI#0083	90237	AFBR070-13	KP112705	Pancas, ES	19° 13' 44'' S	40° 45' 31'' W	133 m
Migonemyia migonei	BOLD:ACD7233	LBMI#0084	90238	AFBR071-13	KP112706	Pancas, ES	19° 13' 44'' S	40° 45' 31'' W	133 m
Migonemyia migonei	BOLD:ACD7233	LBMI#0085	90239	AFBR072-13	KP112707	Pancas, ES	19° 13' 44'' S	40° 45' 31'' W	133 m
Migonemyia migonei	BOLD:ACD7233	LBMI#0086	90240	AFBR073-13	KP112708	Pancas, ES	19° 13' 44'' S	40° 45' 31'' W	133 m
Migonemyia migonei	BOLD:ACD7233	LBMI#0087	90241	AFBR074-13	KP112709	Pancas, ES	19° 13' 44'' S	40° 45' 31'' W	133 m
Migonemyia migonei	BOLD:ACD7233	LBMI#0088	90242	AFBR075-13	KP112710	Pancas, ES	19° 13' 44'' S	40° 45' 31'' W	133 m
Migonemyia migonei	BOLD:ACD7233	LBMI#0089	90243	AFBR076-13	KP112711	Pancas, ES	19° 13' 44'' S	40° 45' 31'' W	133 m
Migonemyia migonei	BOLD:ACD7233	LBMI#0174	90331	AFBR314-14	KP112712	Pancas, ES	19° 13' 44'' S	40° 45' 31'' W	133 m
Migonemyia migonei	BOLD:ACD7233	LBMI#0170	90330	AFBR312-14	KP112713	Pancas, ES	19° 13' 44'' S	40° 45' 31'' W	133 m
Migonemyia migonei	BOLD:ACD7233	LBMI#0090	90244	AFBR077-13	KP112714	Pancas, ES	19° 13' 44'' S	40° 45' 31'' W	133 m
Nyssomyia intermedia	BOLD:ACK2790	LBMI#0220	90343	AFBR353-14	KP112715	Pancas, ES	19° 13' 44'' S	40° 45' 31'' W	133 m

Nyssomyia intermedia	BOLD:ACK2790	LBMI#0219	90342	AFBR352-14	KP112716	Pancas, ES	19° 13’ 44’’ S	40° 45’ 31’’ W	133 m
Nyssomyia intermedia	BOLD:ACK2790	LBMI#0254	90411	AFBR377-14	KP112717	Pancas, ES	19° 13’ 44’’ S	40° 45’ 31’’ W	133 m
Nyssomyia intermedia	BOLD:ACK2790	LBMI#0218	90341	AFBR351-14	KP112718	Pancas, ES	19° 13’ 44’’ S	40° 45’ 31’’ W	133 m
Nyssomyia intermedia	BOLD:ACK2790	LBMI#0217	90340	AFBR350-14	KP112719	Pancas, ES	19° 13’ 44’’ S	40° 45’ 31’’ W	133 m
Nyssomyia intermedia	BOLD:ACK2790	LBMI#0216	90339	AFBR349-14	KP112720	Pancas, ES	19° 13’ 44’’ S	40° 45’ 31’’ W	133 m
Nyssomyia intermedia	BOLD:ACK2790	LBMI#0215	90338	AFBR348-14	KP112721	Pancas, ES	19° 13’ 44’’ S	40° 45’ 31’’ W	133 m
Nyssomyia intermedia	BOLD:ACK2790	LBMI#0214	90337	AFBR347-14	KP112722	Pancas, ES	19° 13’ 44’’ S	40° 45’ 31’’ W	133 m
Nyssomyia intermedia	BOLD:ACK2790	LBMI#0213	90336	AFBR346-14	KP112723	Pancas, ES	19° 13’ 44’’ S	40° 45’ 31’’ W	133 m
Nyssomyia intermedia	BOLD:ACK2790	LBMI#0212	90335	AFBR345-14	KP112724	Pancas, ES	19° 13’ 44’’ S	40° 45’ 31’’ W	133 m
Nyssomyia intermedia	BOLD:ACK2790	LBMI#0209	90332	AFBR343-14	KP112725	Pancas, ES	19° 13’ 44’’ S	40° 45’ 31’’ W	133 m
Nyssomyia intermedia	BOLD:ACK2790	LBMI#0157	90313	AFBR305-14	KP112726	Pancas, ES	19° 13’ 44’’ S	40° 45’ 31’’ W	133 m
Nyssomyia intermedia	BOLD:ACK2790	LBMI#0153	90312	AFBR301-14	KP112727	Pancas, ES	19° 13’ 44’’ S	40° 45’ 31’’ W	133 m
Nyssomyia intermedia	BOLD:ACK2790	LBMI#0152	90311	AFBR300-14	KP112728	Pancas, ES	19° 13’ 44’’ S	40° 45’ 31’’ W	133 m
Nyssomyia intermedia	BOLD:ACK2790	LBMI#0141	90310	AFBR291-14	KP112729	Pancas, ES	19° 13’ 44’’ S	40° 45’ 31’’ W	133 m
Nyssomyia intermedia	BOLD:ACK2790	LBMI#0527	90615	AFBR731-14	KP112730	Santa Leopoldina, ES	20° 08’ 16” S	40° 30’ 57 W	51 m
Nyssomyia intermedia	BOLD:ACK2790	LBMI#0120	90314	AFBR271-14	KP112731	Pancas, ES	19° 13’ 44’’ S	40° 45’ 31’’ W	133 m
Nyssomyia intermedia	BOLD:ACK2790	LBMI#0105	90261	AFBR256-14	KP112732	Pancas, ES	19° 13’ 44’’ S	40° 45’ 31’’ W	133 m
Nyssomyia intermedia	BOLD:ACK2790	LBMI#0104	90260	AFBR255-14	KP112733	Pancas, ES	19° 13’ 44’’ S	40° 45’ 31’’ W	133 m
Nyssomyia intermedia	BOLD:ACK2790	LBMI#0055	90259	AFBR048-13	KP112734	Pancas, ES	19° 13’ 44’’ S	40° 45’ 31’’ W	133 m
Nyssomyia intermedia	BOLD:ACK2790	LBMI#0054	90258	AFBR047-13	KP112735	Pancas, ES	19° 13’ 44’’ S	40° 45’ 31’’ W	133 m
Nyssomyia intermedia	BOLD:ACK2790	LBMI#0053	90257	AFBR046-13	KP112736	Pancas, ES	19° 13’ 44’’ S	40° 45’ 31’’ W	133 m
Nyssomyia intermedia	BOLD:ACK2790	LBMI#0479	90577	AFBR576-14	KP112737	Santa Leopoldina, ES	20° 08’ 16” S	40° 30’ 57 W	51 m
Nyssomyia intermedia	BOLD:ACK2790	LBMI#0014	90256	AFBR013-13	KP112738	Pancas, ES	19° 13’ 44’’ S	40° 45’ 31’’ W	133 m
Nyssomyia intermedia	BOLD:ACK2790	LBMI#0010	90255	AFBR009-13	KP112739	Pancas, ES	19° 13’ 44’’ S	40° 45’ 31’’ W	133 m
Nyssomyia intermedia	BOLD:ACK2790	LBMI#0009	90254	AFBR008-13	KP112740	Pancas, ES	19° 13’ 44’’ S	40° 45’ 31’’ W	133 m
Nyssomyia intermedia	BOLD:ACK2790	LBMI#0008	90253	AFBR007-13	KP112741	Pancas, ES	19° 13’ 44’’ S	40° 45’ 31’’ W	133 m
Nyssomyia intermedia	BOLD:ACK2790	LBMI#0495	90583	AFBR591-14	KP112742	Santa Leopoldina, ES	20° 08’ 16” S	40° 30’ 57 W	51 m
Nyssomyia intermedia	BOLD:ACK2790	LBMI#0496	90584	AFBR592-14	KP112743	Santa Leopoldina, ES	20° 08’ 16” S	40° 30’ 57 W	51 m
Nyssomyia intermedia	BOLD:ACK2790	LBMI#0497	90585	AFBR593-14	KP112744	Santa Leopoldina, ES	20° 08’ 16” S	40° 30’ 57 W	51 m
Nyssomyia intermedia	BOLD:ACK2790	LBMI#0498	90586	AFBR594-14	KP112745	Santa Leopoldina, ES	20° 08’ 16” S	40° 30’ 57 W	51 m
Nyssomyia intermedia	BOLD:ACK2790	LBMI#0500	90588	AFBR596-14	KP112746	Santa Leopoldina, ES	20° 08’ 16” S	40° 30’ 57 W	51 m
Nyssomyia intermedia	BOLD:ACK2790	LBMI#0501	90589	AFBR597-14	KP112747	Santa Leopoldina, ES	20° 08’ 16” S	40° 30’ 57 W	51 m
Nyssomyia intermedia	BOLD:ACK2790	LBMI#0506	90594	AFBR602-14	KP112748	Santa Leopoldina, ES	20° 08’ 16” S	40° 30’ 57 W	51 m

Nyssomyia intermedia	BOLD:ACK2790	LBMI#0005	90252	AFBR004-13	KP112749	Pancas, ES	19° 13’ 44’’ S	40° 45’ 31’’ W	133 m
Nyssomyia intermedia	BOLD:ACK2790	LBMI#0520	90608	AFBR614-14	KP112750	Santa Leopoldina, ES	20° 08’ 16” S	40° 30’ 57 W	51 m
Nyssomyia intermedia	BOLD:ACK2790	LBMI#0521	90609	AFBR615-14	KP112751	Santa Leopoldina, ES	20° 08’ 16” S	40° 30’ 57 W	51 m
Nyssomyia intermedia	BOLD:ACK2790	LBMI#0522	90610	AFBR616-14	KP112752	Santa Leopoldina, ES	20° 08’ 16” S	40° 30’ 57 W	51 m
Nyssomyia intermedia	BOLD:ACK2790	LBMI#0523	90611	AFBR617-14	KP112753	Santa Leopoldina, ES	20° 08’ 16” S	40° 30’ 57 W	51 m
Nyssomyia intermedia	BOLD:ACK2790	LBMI#0524	90612	AFBR618-14	KP112754	Santa Leopoldina, ES	20° 08’ 16” S	40° 30’ 57 W	51 m
Nyssomyia intermedia	BOLD:ACK2790	LBMI#0525	90613	AFBR619-14	KP112755	Santa Leopoldina, ES	20° 08’ 16” S	40° 30’ 57 W	51 m
Nyssomyia intermedia	BOLD:ACK2790	LBMI#0526	90614	AFBR620-14	KP112756	Santa Leopoldina, ES	20° 08’ 16” S	40° 30’ 57 W	51 m
Nyssomyia intermedia	BOLD:ACK2790	LBMI#0528	90616	AFBR621-14	KP112757	Santa Leopoldina, ES	20° 08’ 16” S	40° 30’ 57 W	51 m
Nyssomyia intermedia	BOLD:ACK2790	LBMI#0529	90617	AFBR622-14	KP112758	Santa Leopoldina, ES	20° 08’ 16” S	40° 30’ 57 W	51 m
Nyssomyia intermedia	BOLD:ACK2790	LBMI#0530	90618	AFBR623-14	KP112759	Santa Leopoldina, ES	20° 08’ 16” S	40° 30’ 57 W	51 m
Nyssomyia intermedia	BOLD:ACK2790	LBMI#0531	90619	AFBR624-14	KP112760	Santa Leopoldina, ES	20° 08’ 16” S	40° 30’ 57 W	51 m
Nyssomyia intermedia	BOLD:ACK2790	LBMI#0532	90620	AFBR625-14	KP112761	Santa Leopoldina, ES	20° 08’ 16” S	40° 30’ 57 W	51 m
Nyssomyia whitmani	BOLD:ACM7054	LBMI#0419	90787	AFBR518-14	KP112770	Cáceres, MT	16° 24’ 08’’ S	57° 29’ 55’’ W	286 m
Nyssomyia whitmani	BOLD:ACM7055	LBMI#0405	90773	AFBR504-14	KP112763	Cáceres, MT	16° 24’ 08’’ S	57° 29’ 55’’ W	286 m
Nyssomyia whitmani	BOLD:ACM7055	LBMI#0424	90792	AFBR523-14	KP112764	Cáceres, MT	16° 24’ 08’’ S	57° 29’ 55’’ W	286 m
Nyssomyia whitmani	BOLD:ACM7055	LBMI#0407	90775	AFBR506-14	KP112765	Cáceres, MT	16° 24’ 08’’ S	57° 29’ 55’’ W	286 m
Nyssomyia whitmani	BOLD:ACM7055	LBMI#0417	90785	AFBR516-14	KP112769	Cáceres, MT	16° 24’ 08’’ S	57° 29’ 55’’ W	286 m
Nyssomyia whitmani	BOLD:ACO2803	LBMI#0415	90783	AFBR514-14	KP112762	Cáceres, MT	16° 24’ 08’’ S	57° 29’ 55’’ W	286 m
Nyssomyia whitmani	BOLD:ACO2803	LBMI#0096	90433	AFBR081-13	KP112766	Iúna, ES	20° 21’ 02” S	41° 43’ 27 W	851 m
Nyssomyia whitmani	BOLD:ACO2803	LBMI#0412	90780	AFBR511-14	KP112767	Cáceres, MT	16° 24’ 08’’ S	57° 29’ 55’’ W	286 m
Nyssomyia whitmani	BOLD:ACO2803	LBMI#0421	90789	AFBR520-14	KP112768	Cáceres, MT	16° 24’ 08’’ S	57° 29’ 55’’ W	286 m
Nyssomyia yuilli yuilli	BOLD:ACM5584	LBMI#0356	90743	AFBR470-14	KP112771	Wenceslau Guimarães, BA	13° 35’ 04’’ S	39° 42’ 32’’ W	455 m
Pintomyia bianchigalatiae	BOLD:ACM6928	LBMI#0608	90696	AFBR682-14	KP112772	Domingos Martins, ES	20° 24’ 01” S	40° 45’ 11’’ W	673 m
Pintomyia bianchigalatiae	BOLD:ACM6928	LBMI#0476	90567	AFBR573-14	KP112773	Mantenópolis, ES	18° 51’ 09” S	41° 03’ 59” W	661 m
Pintomyia bianchigalatiae	BOLD:ACM6928	LBMI#0613	90701	AFBR686-14	KP112774	Domingos Martins, ES	20° 24’ 01” S	40° 45’ 11’’ W	673 m
Pintomyia bianchigalatiae	BOLD:ACM6928	LBMI#0456	90551	AFBR555-14	KP112775	Alto Rio Novo, ES	18° 58’ 35” S	41° 00’ 43’’ W	762 m
Pintomyia fischeri	BOLD:ACK3063	LBMI#0448	90510	AFBR547-14	KP112776	Santa Teresa, ES	19° 54’ 30” S	40° 39’ 25’’ W	754 m
Pintomyia fischeri	BOLD:ACK3063	LBMI#0605	90693	AFBR680-14	KP112777	Domingos Martins, ES	20° 24’ 01” S	40° 45’ 11’’ W	673 m
Pintomyia fischeri	BOLD:ACK3063	LBMI#0017	90262	AFBR016-13	KP112778	Pancas, ES	19° 13’ 44’’ S	40° 45’ 31’’ W	133 m
Pintomyia fischeri	BOLD:ACK3063	LBMI#0031	90195	AFBR027-13	KP112779	Pancas, ES	19° 13’ 44’’ S	40° 45’ 31’’ W	133 m
Pintomyia fischeri	BOLD:ACK3063	LBMI#0475	90566	AFBR572-14	KP112780	Mantenópolis, ES	18° 51’ 09” S	41° 03’ 59” W	661 m
Pintomyia fischeri	BOLD:ACK3063	LBMI#0604	90692	AFBR679-14	KP112782	Domingos Martins, ES	20° 24’ 01” S	40° 45’ 11’’ W	673 m

Pintomyia fischeri	BOLD:ACK3063	LBMI#0032	90196	AFBR028-13	KP112783	Pancas, ES	19° 13' 44'' S	40° 45' 31'' W	133 m
Pintomyia fischeri	BOLD:ACK3063	LBMI#0606	90694	AFBR681-14	KP112784	Domingos Martins, ES	20° 24' 01" S	40° 45' 11'' W	673 m
Pintomyia fischeri	BOLD:ACK3063	LBMI#0609	90697	AFBR683-14	KP112785	Domingos Martins, ES	20° 24' 01" S	40° 45' 11'' W	673 m
Pintomyia fischeri	BOLD:ACK3063	LBMI#0610	90698	AFBR684-14	KP112786	Domingos Martins, ES	20° 24' 01" S	40° 45' 11'' W	673 m
Pintomyia fischeri	BOLD:ACK3063	LBMI#0508	90596	AFBR604-14	KP112787	Santa Leopoldina, ES	20° 08' 16" S	40° 30' 57 W	51 m
Pintomyia fischeri	BOLD:ACK3063	LBMI#0660	90720	AFBR728-14	KP112788	Domingos Martins, ES	20° 24' 01" S	40° 45' 11'' W	673 m
Pintomyia fischeri	BOLD:ACK3063	LBMI#0537	90625	AFBR628-14	KP112789	Marilândia, ES	19° 19' 04" S	40° 31' 01" W	581 m
Pintomyia fischeri	BOLD:ACK3063	LBMI#0612	90700	AFBR685-14	KP112790	Domingos Martins, ES	20° 24' 01" S	40° 45' 11'' W	673 m
Pintomyia fischeri	BOLD:ACK3063	LBMI#0582	90670	AFBR741-14	KP112791	Domingos Martins, ES	20° 24' 01" S	40° 45' 11'' W	673 m
Pintomyia fischeri	BOLD:ACK3063	LBMI#0510	90598	AFBR606-14	KP112792	Santa Leopoldina, ES	20° 08' 16" S	40° 30' 57 W	51 m
Pintomyia fischeri	BOLD:ACK3063	LBMI#0583	90671	AFBR665-14	KP112793	Domingos Martins, ES	20° 24' 01" S	40° 45' 11'' W	673 m
Pintomyia fischeri	BOLD:ACK3063	LBMI#0629	90712	AFBR701-14	KP112795	Domingos Martins, ES	20° 24' 01" S	40° 45' 11'' W	673 m
Pintomyia fischeri	BOLD:ACK3063	LBMI#0518	90606	AFBR612-14	KP112796	Santa Leopoldina, ES	20° 08' 16" S	40° 30' 57 W	51 m
Pintomyia fischeri	BOLD:ACK3063	LBMI#0028	90192	AFBR026-13	KP112797	Pancas, ES	19° 13' 44'' S	40° 45' 31'' W	133 m
Pintomyia fischeri	BOLD:ACK3063	LBMI#0033	90197	AFBR029-13	KP112798	Pancas, ES	19° 13' 44'' S	40° 45' 31'' W	133 m
Pintomyia fischeri	BOLD:ACK3063	LBMI#0519	90607	AFBR613-14	KP112799	Santa Leopoldina, ES	20° 08' 16" S	40° 30' 57 W	51 m
Pintomyia fischeri	BOLD:ACK3063	LBMI#0584	90672	AFBR666-14	KP112800	Domingos Martins, ES	20° 24' 01" S	40° 45' 11'' W	673 m
Pintomyia fischeri	BOLD:ACK3063	LBMI#0458	90569	AFBR557-14	KP112801	Alto Rio Novo, ES	18° 58' 35" S	41° 00' 43" W	762 m
Pintomyia fischeri	BOLD:ACK3063	LBMI#0509	90597	AFBR605-14	KP112802	Santa Leopoldina, ES	20° 08' 16" S	40° 30' 57 W	51 m
Pintomyia fischeri	BOLD:ACK3063	LBMI#0027	90191	AFBR025-13	KP112803	Pancas, ES	19° 13' 44'' S	40° 45' 31'' W	133 m
Pintomyia fischeri	BOLD:ACK3063	LBMI#0107	90267	AFBR258-14	KP112804	Pancas, ES	19° 13' 44'' S	40° 45' 31'' W	133 m
Pintomyia fischeri	BOLD:ACK3063	LBMI#0067	90265	AFBR057-13	KP112805	Pancas, ES	19° 13' 44'' S	40° 45' 31'' W	133 m
Pintomyia fischeri	BOLD:ACK3063	LBMI#0066	90264	AFBR056-13	KP112806	Pancas, ES	19° 13' 44'' S	40° 45' 31'' W	133 m
Pintomyia fischeri	BOLD:ACK3063	LBMI#0034	90198	AFBR030-13	KP112807	Pancas, ES	19° 13' 44'' S	40° 45' 31'' W	133 m
Pintomyia fischeri	BOLD:ACK3063	LBMI#0036	90200	AFBR031-13	KP112808	Pancas, ES	19° 13' 44'' S	40° 45' 31'' W	133 m
Pintomyia fischeri	BOLD:ACK3063	LBMI#0106	90266	AFBR257-14	KP112810	Pancas, ES	19° 13' 44'' S	40° 45' 31'' W	133 m
Pintomyia fischeri	BOLD:ACK3063	LBMI#0449	90511	AFBR548-14	KP112811	Santa Teresa, ES	19° 54' 30" S	40° 39' 25'' W	754 m
Pintomyia fischeri	BOLD:ACM6946	LBMI#0557	90645	AFBR644-14	KP112781	Itaguaçu, ES	19° 44' 13" S	40° 58' 09" W	871 m
Pintomyia fischeri	BOLD:ACM6946	LBMI#0556	90644	AFBR643-14	KP112794	Itaguaçu, ES	19° 44' 13" S	40° 58' 09" W	871 m
Pintomyia fischeri	BOLD:ACM6946	LBMI#0450	90528	AFBR549-14	KP112809	Santa Teresa, ES	19° 54' 30" S	40° 39' 25'' W	754 m
Pintomyia misionensis	BOLD:ACK2926	LBMI#0092	90426	AFBR078-13	KP112812	Ibitirama, ES	20° 28' 41'' S	40° 42' 19'' W	842 m
Pintomyia misionensis	BOLD:ACK2926	LBMI#0103	90438	AFBR254-14	KP112813	Iúna, ES	20° 21' 02" S	41° 43' 27 W	851 m
Pintomyia misionensis	BOLD:ACK2926	LBMI#0102	90437	AFBR253-14	KP112814	Iúna, ES	20° 21' 02" S	41° 43' 27 W	851 m

Pintomyia misionensis	BOLD:ACK2926	LBMI#0572	90660	AFBR739-14	KP112815	Itaguaçu, ES	19° 44’ 13” S	40° 58’ 09” W	871 m
Pintomyia misionensis	BOLD:ACK2926	LBMI#0100	90436	AFBR085-13	KP112816	Iúna, ES	20° 21’ 02” S	41° 43’ 27 W	851 m
Pintomyia misionensis	BOLD:ACK2926	LBMI#0098	90435	AFBR083-13	KP112817	Iúna, ES	20° 21’ 02” S	41° 43’ 27 W	851 m
Pintomyia misionensis	BOLD:ACK2926	LBMI#0097	90434	AFBR082-13	KP112818	Iúna, ES	20° 21’ 02” S	41° 43’ 27 W	851 m
Pintomyia misionensis	BOLD:ACK2926	LBMI#0093	90427	AFBR079-13	KP112819	Ibitirama, ES	20° 28’ 41’’ S	40° 42’ 19’’ W	842 m
Pintomyia misionensis	BOLD:ACK2926	LBMI#0562	90650	AFBR737-14	KP112821	Itaguaçu, ES	19° 44’ 13” S	40° 58’ 09” W	871 m
Pintomyia misionensis	BOLD:ACK2926	LBMI#0570	90658	AFBR655-14	KP112822	Itaguaçu, ES	19° 44’ 13” S	40° 58’ 09” W	871 m
Pintomyia misionensis	BOLD:ACK2926	LBMI#0569	90657	AFBR654-14	KP112823	Itaguaçu, ES	19° 44’ 13” S	40° 58’ 09” W	871 m
Pintomyia misionensis	BOLD:ACK2926	LBMI#0114	90431	AFBR265-14	KP112824	Ibitirama, ES	20° 28’ 41’’ S	40° 42’ 19’’ W	842 m
Pintomyia misionensis	BOLD:ACK2926	LBMI#0113	90430	AFBR264-14	KP112825	Ibitirama, ES	20° 28’ 41’’ S	40° 42’ 19’’ W	842 m
Pintomyia misionensis	BOLD:ACK2926	LBMI#0112	90429	AFBR263-14	KP112826	Ibitirama, ES	20° 28’ 41’’ S	40° 42’ 19’’ W	842 m
Pintomyia misionensis	BOLD:ACK2926	LBMI#0111	90428	AFBR262-14	KP112827	Ibitirama, ES	20° 28’ 41’’ S	40° 42’ 19’’ W	842 m
Pintomyia misionensis	BOLD:ACK2926	LBMI#0563	90651	AFBR649-14	KP112828	Itaguaçu, ES	19° 44’ 13” S	40° 58’ 09” W	871 m
Pintomyia misionensis	BOLD:ACK2926	LBMI#0561	90649	AFBR648-14	KP112829	Itaguaçu, ES	19° 44’ 13” S	40° 58’ 09” W	871 m
Pintomyia misionensis	BOLD:ACK2926	LBMI#0624	90707	AFBR696-14	KP112830	Domingos Martins, ES	20° 24’ 01” S	40° 45’ 11’’ W	673 m
Pintomyia misionensis	BOLD:ACK2926	LBMI#0623	90706	AFBR695-14	KP112831	Domingos Martins, ES	20° 24’ 01” S	40° 45’ 11’’ W	673 m
Pintomyia misionensis	BOLD:ACK2926	LBMI#0560	90648	AFBR647-14	KP112832	Itaguaçu, ES	19° 44’ 13” S	40° 58’ 09” W	871 m
Pintomyia misionensis	BOLD:ACK2926	LBMI#0571	90659	AFBR656-14	KP112833	Itaguaçu, ES	19° 44’ 13” S	40° 58’ 09” W	871 m
Pintomyia misionensis	BOLD:ACK2926	LBMI#0559	90647	AFBR646-14	KP112834	Itaguaçu, ES	19° 44’ 13” S	40° 58’ 09” W	871 m
Pintomyia misionensis	BOLD:ACK2926	LBMI#0558	90646	AFBR645-14	KP112835	Itaguaçu, ES	19° 44’ 13” S	40° 58’ 09” W	871 m
Pintomyia misionensis	BOLD:ACM6931	LBMI#0640	90545	AFBR708-14	KP112820	Domingos Martins, ES	20° 24’ 01” S	40° 45’ 11’’ W	673 m
Pintomyia monticola	BOLD:ACK2220	LBMI#0474	90565	AFBR571-14	KP112836	Mantenópolis, ES	18° 51’ 09” S	41° 03’ 59” W	661 m
Pintomyia monticola	BOLD:ACK2220	LBMI#0547	90635	AFBR636-14	KP112837	Marilândia, ES	19° 19’ 04” S	40° 31’ 01” W	581 m
Pintomyia monticola	BOLD:ACK2220	LBMI#0543	90631	AFBR734-14	KP112839	Marilândia, ES	19° 19’ 04” S	40° 31’ 01” W	581 m
Pintomyia monticola	BOLD:ACK2220	LBMI#0541	90629	AFBR631-14	KP112840	Marilândia, ES	19° 19’ 04” S	40° 31’ 01” W	581 m
Pintomyia monticola	BOLD:ACK2220	LBMI#0651	90553	AFBR719-14	KP112845	Alto Rio Novo, ES	18° 58’ 35” S	41° 00’ 43’’ W	762 m
Pintomyia monticola	BOLD:ACK2220	LBMI#0652	90554	AFBR720-14	KP112846	Alto Rio Novo, ES	18° 58’ 35” S	41° 00’ 43’’ W	762 m
Pintomyia monticola	BOLD:ACK2220	LBMI#0653	90555	AFBR721-14	KP112847	Alto Rio Novo, ES	18° 58’ 35” S	41° 00’ 43’’ W	762 m
Pintomyia monticola	BOLD:ACK2220	LBMI#0099	90439	AFBR084-13	KP112850	Iúna, ES	20° 21’ 02” S	41° 43’ 27 W	851 m
Pintomyia monticola	BOLD:ACK2220	LBMI#0447	90527	AFBR546-14	KP112855	Santa Teresa, ES	19° 54’ 30” S	40° 39’ 25’’ W	754 m
Pintomyia monticola	BOLD:ACK2220	LBMI#0101	90440	AFBR252-14	KP112856	Iúna, ES	20° 21’ 02” S	41° 43’ 27 W	851 m
Pintomyia monticola	BOLD:ACK2220	LBMI#0293	90445	AFBR415-14	KP112857	João Neiva, ES	19° 48’ 07” S	40° 30’ 23” W	632 m
Pintomyia monticola	BOLD:ACK2220	LBMI#0451	90546	AFBR550-14	KP112859	Alto Rio Novo, ES	18° 58’ 35” S	41° 00’ 43’’ W	762 m

Pintomyia monticola	BOLD:ACK2220	LBMI#0452	90547	AFBR551-14	KP112860	Alto Rio Novo, ES	18° 58’ 35” S	41° 00’ 43’’ W	762 m
Pintomyia monticola	BOLD:ACK2220	LBMI#0453	90548	AFBR552-14	KP112861	Alto Rio Novo, ES	18° 58’ 35” S	41° 00’ 43’’ W	762 m
Pintomyia monticola	BOLD:ACK2220	LBMI#0454	90549	AFBR553-14	KP112862	Alto Rio Novo, ES	18° 58’ 35” S	41° 00’ 43’’ W	762 m
Pintomyia monticola	BOLD:ACK2220	LBMI#0455	90550	AFBR554-14	KP112863	Alto Rio Novo, ES	18° 58’ 35” S	41° 00’ 43’’ W	762 m
Pintomyia monticola	BOLD:ACK2220	LBMI#0548	90636	AFBR637-14	KP112868	Marilândia, ES	19° 19’ 04” S	40° 31’ 01” W	581 m
Pintomyia monticola	BOLD:ACK2220	LBMI#0291	90444	AFBR413-14	KP112870	João Neiva, ES	19° 48’ 07” S	40° 30’ 23” W	632 m
Pintomyia monticola	BOLD:ACK2220	LBMI#0546	90634	AFBR635-14	KP112871	Marilândia, ES	19° 19’ 04” S	40° 31’ 01” W	581 m
Pintomyia monticola	BOLD:ACK2220	LBMI#0465	90556	AFBR564-14	KP112872	Mantenópolis, ES	18° 51’ 09” S	41° 03’ 59” W	661 m
Pintomyia monticola	BOLD:ACK2220	LBMI#0466	90557	AFBR565-14	KP112873	Mantenópolis, ES	18° 51’ 09” S	41° 03’ 59” W	661 m
Pintomyia monticola	BOLD:ACK2220	LBMI#0467	90558	AFBR566-14	KP112874	Mantenópolis, ES	18° 51’ 09” S	41° 03’ 59” W	661 m
Pintomyia monticola	BOLD:ACK2220	LBMI#0468	90559	AFBR567-14	KP112875	Mantenópolis, ES	18° 51’ 09” S	41° 03’ 59” W	661 m
Pintomyia monticola	BOLD:ACK2220	LBMI#0469	90560	AFBR568-14	KP112876	Mantenópolis, ES	18° 51’ 09” S	41° 03’ 59” W	661 m
Pintomyia monticola	BOLD:ACK2220	LBMI#0470	90561	AFBR569-14	KP112877	Mantenópolis, ES	18° 51’ 09” S	41° 03’ 59” W	661 m
Pintomyia monticola	BOLD:ACK2220	LBMI#0473	90564	AFBR570-14	KP112878	Mantenópolis, ES	18° 51’ 09” S	41° 03’ 59” W	661 m
Pintomyia monticola	BOLD:ACK2220	LBMI#0545	90633	AFBR634-14	KP112879	Marilândia, ES	19° 19’ 04” S	40° 31’ 01” W	581 m
Pintomyia monticola	BOLD:ACK2220	LBMI#0544	90632	AFBR633-14	KP112880	Marilândia, ES	19° 19’ 04” S	40° 31’ 01” W	581 m
Pintomyia monticola	BOLD:ACK2220	LBMI#0542	90630	AFBR632-14	KP112881	Marilândia, ES	19° 19’ 04” S	40° 31’ 01” W	581 m
Pintomyia monticola	BOLD:ACK7188	LBMI#0308	90457	AFBR429-14	KP112843	Alfredo Chaves, ES	20° 29’ 25” S	40° 57’ 28’’ W	1069 m
Pintomyia monticola	BOLD:ACK7188	LBMI#0309	90458	AFBR430-14	KP112844	Alfredo Chaves, ES	20° 29’ 25” S	40° 57’ 28’’ W	1069 m
Pintomyia monticola	BOLD:ACK7189	LBMI#0602	90690	AFBR677-14	KP112838	Domingos Martins, ES	20° 24’ 01” S	40° 45’ 11’’ W	673 m
Pintomyia monticola	BOLD:ACK7189	LBMI#0594	90682	AFBR746-14	KP112841	Domingos Martins, ES	20° 24’ 01” S	40° 45’ 11’’ W	673 m
Pintomyia monticola	BOLD:ACK7189	LBMI#0593	90681	AFBR745-14	KP112842	Domingos Martins, ES	20° 24’ 01” S	40° 45’ 11’’ W	673 m
Pintomyia monticola	BOLD:ACK7189	LBMI#0656	90716	AFBR724-14	KP112848	Domingos Martins, ES	20° 24’ 01” S	40° 45’ 11’’ W	673 m
Pintomyia monticola	BOLD:ACK7189	LBMI#0657	90717	AFBR725-14	KP112849	Domingos Martins, ES	20° 24’ 01” S	40° 45’ 11’’ W	673 m
Pintomyia monticola	BOLD:ACK7189	LBMI#0443	90529	AFBR542-14	KP112851	Santa Teresa, ES	19° 54’ 30” S	40° 39’ 25’’ W	754 m
Pintomyia monticola	BOLD:ACK7189	LBMI#0444	90530	AFBR543-14	KP112852	Santa Teresa, ES	19° 54’ 30” S	40° 39’ 25’’ W	754 m
Pintomyia monticola	BOLD:ACK7189	LBMI#0349	90496	AFBR467-14	KP112864	Santa Teresa, ES	19° 54’ 30” S	40° 39’ 25’’ W	754 m
Pintomyia monticola	BOLD:ACK7189	LBMI#0658	90718	AFBR726-14	KP112865	Domingos Martins, ES	20° 24’ 01” S	40° 45’ 11’’ W	673 m
Pintomyia monticola	BOLD:ACK7189	LBMI#0592	90680	AFBR671-14	KP112866	Domingos Martins, ES	20° 24’ 01” S	40° 45’ 11’’ W	673 m
Pintomyia monticola	BOLD:ACK7189	LBMI#0601	90689	AFBR676-14	KP112867	Domingos Martins, ES	20° 24’ 01” S	40° 45’ 11’’ W	673 m
Pintomyia monticola	BOLD:ACK7189	LBMI#0659	90719	AFBR727-14	KP112869	Domingos Martins, ES	20° 24’ 01” S	40° 45’ 11’’ W	673 m
Pintomyia monticola	BOLD:ACK7189	LBMI#0426	90507	AFBR525-14	KP112883	Santa Teresa, ES	19° 54’ 30” S	40° 39’ 25’’ W	754 m
Pintomyia monticola	BOLD:ACK7189	LBMI#0595	90683	AFBR672-14	KP112884	Domingos Martins, ES	20° 24’ 01” S	40° 45’ 11’’ W	673 m

Pintomyia monticola	BOLD:ACK7190	LBMI#0445	90508	AFBR544-14	KP112853	Santa Teresa, ES	19° 54’ 30” S	40° 39’ 25’’ W	754 m
Pintomyia monticola	BOLD:ACK7190	LBMI#0446	90509	AFBR545-14	KP112854	Santa Teresa, ES	19° 54’ 30” S	40° 39’ 25’’ W	754 m
Pintomyia monticola	BOLD:ACK7190	LBMI#0350	90497	AFBR468-14	KP112858	Santa Teresa, ES	19° 54’ 30” S	40° 39’ 25’’ W	754 m
Pintomyia monticola	BOLD:ACK7190	LBMI#0425	90506	AFBR524-14	KP112882	Santa Teresa, ES	19° 54’ 30” S	40° 39’ 25’’ W	754 m
Pressatia choti	BOLD:ACJ9593	LBMI#0123	90276	AFBR274-14	KP112885	Pancas, ES	19° 13’ 44’’ S	40° 45’ 31’’ W	133 m
Pressatia choti	BOLD:ACJ9593	LBMI#0122	90269	AFBR273-14	KP112886	Pancas, ES	19° 13’ 44’’ S	40° 45’ 31’’ W	133 m
Pressatia choti	BOLD:ACJ9593	LBMI#0121	90284	AFBR272-14	KP112887	Pancas, ES	19° 13’ 44’’ S	40° 45’ 31’’ W	133 m
Pressatia choti	BOLD:ACJ9593	LBMI#0080	90283	AFBR067-13	KP112888	Pancas, ES	19° 13’ 44’’ S	40° 45’ 31’’ W	133 m
Pressatia choti	BOLD:ACJ9593	LBMI#0079	90282	AFBR066-13	KP112889	Pancas, ES	19° 13’ 44’’ S	40° 45’ 31’’ W	133 m
Pressatia choti	BOLD:ACJ9593	LBMI#0078	90281	AFBR065-13	KP112890	Pancas, ES	19° 13’ 44’’ S	40° 45’ 31’’ W	133 m
Pressatia choti	BOLD:ACJ9593	LBMI#0077	90280	AFBR064-13	KP112891	Pancas, ES	19° 13’ 44’’ S	40° 45’ 31’’ W	133 m
Pressatia choti	BOLD:ACJ9593	LBMI#0076	90279	AFBR063-13	KP112892	Pancas, ES	19° 13’ 44’’ S	40° 45’ 31’’ W	133 m
Pressatia choti	BOLD:ACJ9593	LBMI#0074	90277	AFBR062-13	KP112893	Pancas, ES	19° 13’ 44’’ S	40° 45’ 31’’ W	133 m
Pressatia choti	BOLD:ACJ9593	LBMI#0020	90275	AFBR019-13	KP112894	Pancas, ES	19° 13’ 44’’ S	40° 45’ 31’’ W	133 m
Pressatia choti	BOLD:ACJ9593	LBMI#0019	90274	AFBR018-13	KP112895	Pancas, ES	19° 13’ 44’’ S	40° 45’ 31’’ W	133 m
Pressatia choti	BOLD:ACJ9593	LBMI#0015	90273	AFBR014-13	KP112896	Pancas, ES	19° 13’ 44’’ S	40° 45’ 31’’ W	133 m
Pressatia choti	BOLD:ACJ9593	LBMI#0012	90272	AFBR011-13	KP112897	Pancas, ES	19° 13’ 44’’ S	40° 45’ 31’’ W	133 m
Pressatia choti	BOLD:ACJ9593	LBMI#0007	90271	AFBR006-13	KP112898	Pancas, ES	19° 13’ 44’’ S	40° 45’ 31’’ W	133 m
Pressatia choti	BOLD:ACJ9593	LBMI#0001	90268	AFBR001-13	KP112899	Pancas, ES	19° 13’ 44’’ S	40° 45’ 31’’ W	133 m
Pressatia choti	BOLD:ACJ9593	LBMI#0006	90270	AFBR005-13	KP112900	Pancas, ES	19° 13’ 44’’ S	40° 45’ 31’’ W	133 m
Pressatia choti	BOLD:ACJ9593	LBMI#0124	90278	AFBR275-14	KP112901	Pancas, ES	19° 13’ 44’’ S	40° 45’ 31’’ W	133 m
Pressatia sp.	BOLD:ACJ9593	LBMI#0175	90345	AFBR315-14	KP112902	Pancas, ES	19° 13’ 44’’ S	40° 45’ 31’’ W	133 m
Pressatia sp.	BOLD:ACJ9593	LBMI#0251	90405	AFBR374-14	KP112903	Pancas, ES	19° 13’ 44’’ S	40° 45’ 31’’ W	133 m
Pressatia sp.	BOLD:ACJ9593	LBMI#0250	90404	AFBR373-14	KP112904	Pancas, ES	19° 13’ 44’’ S	40° 45’ 31’’ W	133 m
Pressatia sp.	BOLD:ACJ9593	LBMI#0226	90367	AFBR354-14	KP112905	Pancas, ES	19° 13’ 44’’ S	40° 45’ 31’’ W	133 m
Pressatia sp.	BOLD:ACJ9593	LBMI#0228	90368	AFBR356-14	KP112906	Pancas, ES	19° 13’ 44’’ S	40° 45’ 31’’ W	133 m
Pressatia sp.	BOLD:ACJ9593	LBMI#0188	90347	AFBR325-14	KP112907	Pancas, ES	19° 13’ 44’’ S	40° 45’ 31’’ W	133 m
Pressatia sp.	BOLD:ACJ9593	LBMI#0184	90346	AFBR321-14	KP112908	Pancas, ES	19° 13’ 44’’ S	40° 45’ 31’’ W	133 m
Pressatia sp.	BOLD:ACJ9593	LBMI#0166	90344	AFBR309-14	KP112909	Pancas, ES	19° 13’ 44’’ S	40° 45’ 31’’ W	133 m
Psathyromyia bigeniculata	BOLD:ACM6710	LBMI#0404	90772	AFBR503-14	KP112911	Cáceres, MT	16° 24’ 08’’ S	57° 29’ 55’’ W	286 m
Psathyromyia bigeniculata	BOLD:ACM6710	LBMI#0402	90770	AFBR501-14	KP112912	Cáceres, MT	16° 24’ 08’’ S	57° 29’ 55’’ W	286 m
Psathyromyia bigeniculata	BOLD:ACM6810	LBMI#0504	90592	AFBR600-14	KP112910	Santa Leopoldina, ES	20° 08’ 16” S	40° 30’ 57 W	51 m
Psathyromyia bigeniculata	BOLD:ACM6810	LBMI#0505	90593	AFBR601-14	KP112913	Santa Leopoldina, ES	20° 08’ 16” S	40° 30’ 57 W	51 m

Psathyromyia bigeniculata	BOLD:ACM6810	LBMI#0499	90587	AFBR595-14	KP112914	Santa Leopoldina, ES	20° 08’ 16” S	40° 30’ 57 W	51 m
Psathyromyia bigeniculata	BOLD:ACM6810	LBMI#0507	90595	AFBR603-14	KP112915	Santa Leopoldina, ES	20° 08’ 16” S	40° 30’ 57 W	51 m
Psathyromyia bigeniculata	BOLD:ACM6810	LBMI#0502	90590	AFBR598-14	KP112916	Santa Leopoldina, ES	20° 08’ 16” S	40° 30’ 57 W	51 m
Psathyromyia bigeniculata	BOLD:ACM6810	LBMI#0503	90591	AFBR599-14	KP112917	Santa Leopoldina, ES	20° 08’ 16” S	40° 30’ 57 W	51 m
Psathyromyia limai	BOLD:ACK6674	LBMI#0553	90641	AFBR642-14	KP112918	Itaguaçu, ES	19° 44’ 13” S	40° 58’ 09” W	871 m
Psathyromyia limai	BOLD:ACK6674	LBMI#0539	90627	AFBR630-14	KP112919	Marilândia, ES	19° 19’ 04” S	40° 31’ 01” W	581 m
Psathyromyia limai	BOLD:ACK6674	LBMI#0325	90467	AFBR444-14	KP112920	Santa Maria de Jetibá, ES	19° 58’ 54” S	40° 48’ 46” W	844 m
Psathyromyia limai	BOLD:ACK6674	LBMI#0310	90490	AFBR431-14	KP112921	Alfredo Chaves, ES	20° 29’ 25” S	40° 57’ 28’’ W	1069 m
Psathyromyia limai	BOLD:ACK6674	LBMI#0326	90468	AFBR445-14	KP112922	Santa Maria de Jetibá, ES	19° 58’ 54” S	40° 48’ 46” W	844 m
Psathyromyia limai	BOLD:ACK6674	LBMI#0627	90710	AFBR699-14	KP112923	Domingos Martins, ES	20° 24’ 01” S	40° 45’ 11’’ W	673 m
Psathyromyia limai	BOLD:ACK6674	LBMI#0290	90447	AFBR412-14	KP112924	João Neiva, ES	19° 48’ 07” S	40° 30’ 23” W	632 m
Psathyromyia limai	BOLD:ACK6674	LBMI#0564	90652	AFBR650-14	KP112925	Itaguaçu, ES	19° 44’ 13” S	40° 58’ 09” W	871 m
Psathyromyia limai	BOLD:ACK6674	LBMI#0555	90643	AFBR736-14	KP112926	Itaguaçu, ES	19° 44’ 13” S	40° 58’ 09” W	871 m
Psathyromyia limai	BOLD:ACK6674	LBMI#0554	90642	AFBR735-14	KP112927	Itaguaçu, ES	19° 44’ 13” S	40° 58’ 09” W	871 m
Psathyromyia limai	BOLD:ACK6674	LBMI#0324	90466	AFBR443-14	KP112928	Santa Maria de Jetibá, ES	19° 58’ 54” S	40° 48’ 46” W	844 m
Psathyromyia limai	BOLD:ACK6674	LBMI#0628	90711	AFBR700-14	KP112929	Domingos Martins, ES	20° 24’ 01” S	40° 45’ 11’’ W	673 m
Psathyromyia limai	BOLD:ACK6674	LBMI#0540	90628	AFBR733-14	KP112930	Marilândia, ES	19° 19’ 04” S	40° 31’ 01” W	581 m
Psathyromyia limai	BOLD:ACK6674	LBMI#0289	90446	AFBR411-14	KP112931	João Neiva, ES	19° 48’ 07” S	40° 30’ 23” W	632 m
Psathyromyia limai	BOLD:ACK6674	LBMI#0551	90639	AFBR640-14	KP112932	Santa Maria de Jetibá, ES	19° 58’ 54” S	40° 48’ 46” W	844 m
Psathyromyia limai	BOLD:ACK6674	LBMI#0552	90640	AFBR641-14	KP112933	Itaguaçu, ES	19° 44’ 13” S	40° 58’ 09” W	871 m
Psathyromyia limai	BOLD:ACK6674	LBMI#0313	90469	AFBR434-14	KP112934	Alfredo Chaves, ES	20° 29’ 25” S	40° 57’ 28’’ W	1069 m
Psathyromyia limai	BOLD:ACK6674	LBMI#0314	90491	AFBR435-14	KP112935	Alfredo Chaves, ES	20° 29’ 25” S	40° 57’ 28’’ W	1069 m
Psathyromyia limai	BOLD:ACK6674	LBMI#0315	90492	AFBR436-14	KP112936	Alfredo Chaves, ES	20° 29’ 25” S	40° 57’ 28’’ W	1069 m
Psathyromyia limai	BOLD:ACK6674	LBMI#0316	90465	AFBR437-14	KP112937	Alfredo Chaves, ES	20° 29’ 25” S	40° 57’ 28’’ W	1069 m
Psathyromyia limai	BOLD:ACK6674	LBMI#0312	90464	AFBR433-14	KP112938	Alfredo Chaves, ES	20° 29’ 25” S	40° 57’ 28’’ W	1069 m
Psathyromyia lutziana	BOLD:ACK4671	LBMI#0198	90369	AFBR333-14	KP112939	Pancas, ES	19° 13’ 44’’ S	40° 45’ 31’’ W	133 m
Psathyromyia lutziana	BOLD:ACK4671	LBMI#0181	90348	AFBR318-14	KP112940	Pancas, ES	19° 13’ 44’’ S	40° 45’ 31’’ W	133 m
Psathyromyia pascalei	BOLD:ACK5205	LBMI#0305	90459	AFBR426-14	KP112941	Alfredo Chaves, ES	20° 29’ 25” S	40° 57’ 28’’ W	1069 m
Psathyromyia pascalei	BOLD:ACK5205	LBMI#0266	90423	AFBR389-14	KP112942	Pancas, ES	19° 13’ 44’’ S	40° 45’ 31’’ W	133 m
Psathyromyia pascalei	BOLD:ACK5205	LBMI#0463	90574	AFBR562-14	KP112943	Alto Rio Novo, ES	18° 58’ 35” S	41° 00’ 43’’ W	762 m
Psathyromyia pascalei	BOLD:ACK5205	LBMI#0462	90573	AFBR561-14	KP112944	Alto Rio Novo, ES	18° 58’ 35” S	41° 00’ 43’’ W	762 m
Psathyromyia pascalei	BOLD:ACK5205	LBMI#0461	90572	AFBR560-14	KP112945	Alto Rio Novo, ES	18° 58’ 35” S	41° 00’ 43’’ W	762 m
Psathyromyia pascalei	BOLD:ACK5205	LBMI#0457	90552	AFBR556-14	KP112946	Alto Rio Novo, ES	18° 58’ 35” S	41° 00’ 43’’ W	762 m

Psathyromyia pascalei	BOLD:ACK5205	LBMI#0625	90708	AFBR697-14	KP112947	Domingos Martins, ES	20° 24’ 01” S	40° 45’ 11’’ W	673 m
Psathyromyia pascalei	BOLD:ACK5205	LBMI#0258	90412	AFBR381-14	KP112948	Pancas, ES	19° 13’ 44’’ S	40° 45’ 31’’ W	133 m
Psathyromyia pascalei	BOLD:ACK5205	LBMI#0635	90730	AFBR749-14	KP112949	Domingos Martins, ES	20° 24’ 01” S	40° 45’ 11’’ W	673 m
Psathyromyia pascalei	BOLD:ACK5205	LBMI#0386	90732	AFBR489-14	KP112950	Bom Jesus do Itabapoana, ES	21° 03’ 25’’ S	41° 47’ 31’’ W	500 m
Psathyromyia pascalei	BOLD:ACK5205	LBMI#0626	90709	AFBR698-14	KP112951	Domingos Martins, ES	20° 24’ 01” S	40° 45’ 11’’ W	673 m
Psathyromyia pascalei	BOLD:ACK5205	LBMI#0534	90622	AFBR626-14	KP112952	Baixo Guandu, ES	19° 21’ 04” S	40° 49’ 48” W	719 m
Psathyromyia pascalei	BOLD:ACK5205	LBMI#0533	90621	AFBR732-14	KP112953	Baixo Guandu, ES	19° 21’ 04” S	40° 49’ 48” W	719 m
Psathyromyia pascalei	BOLD:ACK5205	LBMI#0304	90493	AFBR425-14	KP112954	Alfredo Chaves, ES	20° 29’ 25” S	40° 57’ 28’’ W	1069 m
Psathyromyia pascalei	BOLD:ACK5205	LBMI#0634	90729	AFBR748-14	KP112955	Domingos Martins, ES	20° 24’ 01” S	40° 45’ 11’’ W	673 m
Psathyromyia pascalei	BOLD:ACK5205	LBMI#0307	90461	AFBR428-14	KP112956	Alfredo Chaves, ES	20° 29’ 25” S	40° 57’ 28’’ W	1069 m
Psathyromyia pascalei	BOLD:ACK5205	LBMI#0179	90349	AFBR316-14	KP112957	Pancas, ES	19° 13’ 44’’ S	40° 45’ 31’’ W	133 m
Psathyromyia pascalei	BOLD:ACK5205	LBMI#0311	90463	AFBR432-14	KP112958	Alfredo Chaves, ES	20° 29’ 25” S	40° 57’ 28’’ W	1069 m
Psathyromyia pascalei	BOLD:ACK5205	LBMI#0327	90462	AFBR446-14	KP112959	Santa Maria de Jetibá, ES	19° 58’ 54” S	40° 48’ 46” W	844 m
Psathyromyia pelloni	BOLD:ACK6607	LBMI#0288	90448	AFBR410-14	KP112960	João Neiva, ES	19° 48’ 07” S	40° 30’ 23” W	632 m
Psychodopygus ayrozai	BOLD:ACM6740	LBMI#0599	90687	AFBR674-14	KP112961	Domingos Martins, ES	20° 24’ 01” S	40° 45’ 11’’ W	673 m
Psychodopygus ayrozai	BOLD:ACM6740	LBMI#0616	90721	AFBR689-14	KP112962	Domingos Martins, ES	20° 24’ 01” S	40° 45’ 11’’ W	673 m
Psychodopygus ayrozai	BOLD:ACM6740	LBMI#0620	90725	AFBR692-14	KP112963	Domingos Martins, ES	20° 24’ 01” S	40° 45’ 11’’ W	673 m
Psychodopygus ayrozai	BOLD:ACM6740	LBMI#0618	90723	AFBR691-14	KP112964	Domingos Martins, ES	20° 24’ 01” S	40° 45’ 11’’ W	673 m
Psychodopygus ayrozai	BOLD:ACM6740	LBMI#0600	90688	AFBR675-14	KP112965	Domingos Martins, ES	20° 24’ 01” S	40° 45’ 11’’ W	673 m
Psychodopygus ayrozai	BOLD:ACM6740	LBMI#0622	90705	AFBR694-14	KP112966	Domingos Martins, ES	20° 24’ 01” S	40° 45’ 11’’ W	673 m
Psychodopygus ayrozai	BOLD:ACM6740	LBMI#0378	90.754	AFBR750-14	KP112967	Wenceslau Guimarães, BA	13° 35’ 04’’ S	39° 42’ 32’’ W	455 m
Psychodopygus davisi	BOLD:ACM6736	LBMI#0516	90604	AFBR610-14	KP112968	Santa Leopoldina, ES	20° 08’ 16” S	40° 30’ 57 W	51 m
Psychodopygus davisi	BOLD:ACM6736	LBMI#0511	90599	AFBR607-14	KP112969	Santa Leopoldina, ES	20° 08’ 16” S	40° 30’ 57 W	51 m
Psychodopygus davisi	BOLD:ACM6736	LBMI#0512	90600	AFBR608-14	KP112970	Santa Leopoldina, ES	20° 08’ 16” S	40° 30’ 57 W	51 m
Psychodopygus davisi	BOLD:ACM6736	LBMI#0515	90603	AFBR609-14	KP112971	Santa Leopoldina, ES	20° 08’ 16” S	40° 30’ 57 W	51 m
Psychodopygus davisi	BOLD:ACM6736	LBMI#0517	90605	AFBR611-14	KP112972	Santa Leopoldina, ES	20° 08’ 16” S	40° 30’ 57 W	51 m
Psychodopygus davisi	BOLD:ACM6736	LBMI#0513	90601	AFBR729-14	KP112973	Santa Leopoldina, ES	20° 08’ 16” S	40° 30’ 57 W	51 m
Psychodopygus davisi	BOLD:ACM6736	LBMI#0514	90602	AFBR730-14	KP112974	Santa Leopoldina, ES	20° 08’ 16” S	40° 30’ 57 W	51 m
Psychodopygus hirsutus	BOLD:ACK6158	LBMI#0480	90578	AFBR577-14	KP112975	Santa Leopoldina, ES	20° 08’ 16” S	40° 30’ 57 W	51 m
Psychodopygus hirsutus	BOLD:ACK6158	LBMI#0596	90684	AFBR747-14	KP112976	Domingos Martins, ES	20° 24’ 01” S	40° 45’ 11’’ W	673 m
Psychodopygus hirsutus	BOLD:ACK6158	LBMI#0271	90424	AFBR394-14	KP112977	Pancas, ES	19° 13’ 44’’ S	40° 45’ 31’’ W	133 m
Psychodopygus hirsutus	BOLD:ACK6158	LBMI#0272	90425	AFBR395-14	KP112978	Pancas, ES	19° 13’ 44’’ S	40° 45’ 31’’ W	133 m
Psychodopygus hirsutus	BOLD:ACK6158	LBMI#0591	90679	AFBR744-14	KP112979	Domingos Martins, ES	20° 24’ 01” S	40° 45’ 11’’ W	673 m

Psychodopygus hirsutus	BOLD:ACK6158	LBMI#0590	90678	AFBR743-14	KP112980	Domingos Martins, ES	20° 24’ 01’’ S	40° 45’ 11’’ W	673 m
Psychodopygus hirsutus	BOLD:ACK6158	LBMI#0589	90677	AFBR742-14	KP112981	Domingos Martins, ES	20° 24’ 01’’ S	40° 45’ 11’’ W	673 m
Psychodopygus hirsutus	BOLD:ACK6158	LBMI#0273	90385	AFBR396-14	KP112982	Pancas, ES	19° 13’ 44’’ S	40° 45’ 31’’ W	133 m
Psychodopygus hirsutus	BOLD:ACK6158	LBMI#0294	90449	AFBR416-14	KP112983	João Neiva, ES	19° 48’ 07” S	40° 30’ 23” W	632 m
Psychodopygus hirsutus	BOLD:ACK6158	LBMI#0295	90450	AFBR417-14	KP112984	João Neiva, ES	19° 48’ 07” S	40° 30’ 23” W	632 m
Psychodopygus hirsutus	BOLD:ACK6158	LBMI#0296	90451	AFBR418-14	KP112985	João Neiva, ES	19° 48’ 07” S	40° 30’ 23” W	632 m
Psychodopygus hirsutus	BOLD:ACK6158	LBMI#0297	90452	AFBR419-14	KP112986	João Neiva, ES	19° 48’ 07” S	40° 30’ 23” W	632 m
Psychodopygus hirsutus	BOLD:ACK6158	LBMI#0614	90702	AFBR687-14	KP112987	Domingos Martins, ES	20° 24’ 01’’ S	40° 45’ 11’’ W	673 m
Psychodopygus hirsutus	BOLD:ACK6158	LBMI#0615	90703	AFBR688-14	KP112988	Domingos Martins, ES	20° 24’ 01’’ S	40° 45’ 11’’ W	673 m
Psychodopygus hirsutus	BOLD:ACK6158	LBMI#0478	90576	AFBR575-14	KP112989	Santa Leopoldina, ES	20° 08’ 16” S	40° 30’ 57 W	51 m
Psychodopygus hirsutus	BOLD:ACK6158	LBMI#0617	90722	AFBR690-14	KP112990	Domingos Martins, ES	20° 24’ 01’’ S	40° 45’ 11’’ W	673 m
Psychodopygus hirsutus	BOLD:ACK6158	LBMI#0588	90676	AFBR670-14	KP112991	Domingos Martins, ES	20° 24’ 01’’ S	40° 45’ 11’’ W	673 m
Psychodopygus hirsutus	BOLD:ACK6158	LBMI#0586	90674	AFBR668-14	KP112992	Domingos Martins, ES	20° 24’ 01’’ S	40° 45’ 11’’ W	673 m
Psychodopygus hirsutus	BOLD:ACK6158	LBMI#0621	90704	AFBR693-14	KP112993	Domingos Martins, ES	20° 24’ 01’’ S	40° 45’ 11’’ W	673 m
Psychodopygus hirsutus	BOLD:ACK6158	LBMI#0587	90675	AFBR669-14	KP112994	Domingos Martins, ES	20° 24’ 01’’ S	40° 45’ 11’’ W	673 m
Psychodopygus hirsutus	BOLD:ACK6158	LBMI#0598	90686	AFBR673-14	KP112995	Domingos Martins, ES	20° 24’ 01’’ S	40° 45’ 11’’ W	673 m
Psychodopygus hirsutus	BOLD:ACK6158	LBMI#0492	90580	AFBR588-14	KP112996	Santa Leopoldina, ES	20° 08’ 16” S	40° 30’ 57 W	51 m
Psychodopygus hirsutus	BOLD:ACK6158	LBMI#0298	90453	AFBR420-14	KP112997	João Neiva, ES	19° 48’ 07” S	40° 30’ 23” W	632 m
Psychodopygus hirsutus	BOLD:ACK6158	LBMI#0655	90715	AFBR723-14	KP112998	Domingos Martins, ES	20° 24’ 01’’ S	40° 45’ 11’’ W	673 m
Psychodopygus hirsutus	BOLD:ACK6158	LBMI#0654	90714	AFBR722-14	KP112999	Domingos Martins, ES	20° 24’ 01’’ S	40° 45’ 11’’ W	673 m
Psychodopygus matosi	BOLD:ACK6257	LBMI#0646	90536	AFBR714-14	KP113000	Santa Teresa, ES	19° 54’ 30” S	40° 39’ 25’’ W	754 m
Psychodopygus matosi	BOLD:ACK6257	LBMI#0647	90537	AFBR715-14	KP113001	Santa Teresa, ES	19° 54’ 30” S	40° 39’ 25’’ W	754 m
Psychodopygus matosi	BOLD:ACK6257	LBMI#0648	90538	AFBR716-14	KP113002	Santa Teresa, ES	19° 54’ 30” S	40° 39’ 25’’ W	754 m
Psychodopygus matosi	BOLD:ACK6257	LBMI#0649	90539	AFBR717-14	KP113003	Santa Teresa, ES	19° 54’ 30” S	40° 39’ 25’’ W	754 m
Psychodopygus matosi	BOLD:ACK6257	LBMI#0650	90540	AFBR718-14	KP113004	Santa Teresa, ES	19° 54’ 30” S	40° 39’ 25’’ W	754 m
Psychodopygus matosi	BOLD:ACK6257	LBMI#0580	90668	AFBR663-14	KP113005	Afonso Cláudio, ES	20° 12’ 53’’ S	41° 02’ 31’’ W	1030 m
Psychodopygus matosi	BOLD:ACK6257	LBMI#0578	90666	AFBR661-14	KP113006	Afonso Cláudio, ES	20° 12’ 53’’ S	41° 02’ 31’’ W	1030 m
Psychodopygus matosi	BOLD:ACK6257	LBMI#0576	90664	AFBR660-14	KP113007	Afonso Cláudio, ES	20° 12’ 53’’ S	41° 02’ 31’’ W	1030 m
Psychodopygus matosi	BOLD:ACK6257	LBMI#0575	90663	AFBR659-14	KP113008	Afonso Cláudio, ES	20° 12’ 53’’ S	41° 02’ 31’’ W	1030 m
Psychodopygus matosi	BOLD:ACK6257	LBMI#0573	90661	AFBR657-14	KP113010	Afonso Cláudio, ES	20° 12’ 53’’ S	41° 02’ 31’’ W	1030 m
Psychodopygus matosi	BOLD:ACK6257	LBMI#0536	90624	AFBR627-14	KP113011	Marilândia, ES	19° 19’ 04” S	40° 31’ 01” W	581 m
Psychodopygus matosi	BOLD:ACK6257	LBMI#0440	90524	AFBR539-14	KP113012	Santa Teresa, ES	19° 54’ 30” S	40° 39’ 25’’ W	754 m
Psychodopygus matosi	BOLD:ACK6257	LBMI#0439	90523	AFBR538-14	KP113013	Santa Teresa, ES	19° 54’ 30” S	40° 39’ 25’’ W	754 m

Psychodopygus matosi	BOLD:ACK6257	LBMI#0438	90522	AFBR537-14	KP113014	Santa Teresa, ES	19° 54’ 30” S	40° 39’ 25’’ W	754 m
Psychodopygus matosi	BOLD:ACK6257	LBMI#0437	90521	AFBR536-14	KP113015	Santa Teresa, ES	19° 54’ 30” S	40° 39’ 25’’ W	754 m
Psychodopygus matosi	BOLD:ACK6257	LBMI#0436	90520	AFBR535-14	KP113016	Santa Teresa, ES	19° 54’ 30” S	40° 39’ 25’’ W	754 m
Psychodopygus matosi	BOLD:ACK6257	LBMI#0435	90519	AFBR534-14	KP113017	Santa Teresa, ES	19° 54’ 30” S	40° 39’ 25’’ W	754 m
Psychodopygus matosi	BOLD:ACK6257	LBMI#0434	90518	AFBR533-14	KP113018	Santa Teresa, ES	19° 54’ 30” S	40° 39’ 25’’ W	754 m
Psychodopygus matosi	BOLD:ACK6257	LBMI#0433	90517	AFBR532-14	KP113019	Santa Teresa, ES	19° 54’ 30” S	40° 39’ 25’’ W	754 m
Psychodopygus matosi	BOLD:ACK6257	LBMI#0432	90516	AFBR531-14	KP113020	Santa Teresa, ES	19° 54’ 30” S	40° 39’ 25’’ W	754 m
Psychodopygus matosi	BOLD:ACK6257	LBMI#0431	90515	AFBR530-14	KP113021	Santa Teresa, ES	19° 54’ 30” S	40° 39’ 25’’ W	754 m
Psychodopygus matosi	BOLD:ACK6257	LBMI#0429	90514	AFBR528-14	KP113022	Santa Teresa, ES	19° 54’ 30” S	40° 39’ 25’’ W	754 m
Psychodopygus matosi	BOLD:ACK6257	LBMI#0428	90513	AFBR527-14	KP113023	Santa Teresa, ES	19° 54’ 30” S	40° 39’ 25’’ W	754 m
Psychodopygus matosi	BOLD:ACK6257	LBMI#0427	90512	AFBR526-14	KP113024	Santa Teresa, ES	19° 54’ 30” S	40° 39’ 25’’ W	754 m
Psychodopygus matosi	BOLD:ACK6257	LBMI#0641	90531	AFBR709-14	KP113025	Santa Teresa, ES	19° 54’ 30” S	40° 39’ 25’’ W	754 m
Psychodopygus matosi	BOLD:ACK6257	LBMI#0642	90532	AFBR710-14	KP113026	Santa Teresa, ES	19° 54’ 30” S	40° 39’ 25’’ W	754 m
Psychodopygus matosi	BOLD:ACK6257	LBMI#0643	90533	AFBR711-14	KP113027	Santa Teresa, ES	19° 54’ 30” S	40° 39’ 25’’ W	754 m
Psychodopygus matosi	BOLD:ACK6257	LBMI#0644	90534	AFBR712-14	KP113028	Santa Teresa, ES	19° 54’ 30” S	40° 39’ 25’’ W	754 m
Psychodopygus matosi	BOLD:ACK6257	LBMI#0645	90535	AFBR713-14	KP113029	Santa Teresa, ES	19° 54’ 30” S	40° 39’ 25’’ W	754 m
Psychodopygus matosi	BOLD:ACK6257	LBMI#0346	90499	AFBR465-14	KP113030	Santa Teresa, ES	19° 54’ 30” S	40° 39’ 25’’ W	754 m
Psychodopygus matosi	BOLD:ACK6257	LBMI#0345	90498	AFBR464-14	KP113031	Santa Teresa, ES	19° 54’ 30” S	40° 39’ 25’’ W	754 m
Psychodopygus matosi	BOLD:ACK6257	LBMI#0318	90455	AFBR439-14	KP113032	Santa Maria de Jetibá, ES	19° 58’ 54” S	40° 48’ 46” W	844 m
Psychodopygus matosi	BOLD:ACK6257	LBMI#0317	90454	AFBR438-14	KP113033	Santa Maria de Jetibá, ES	19° 58’ 54 S	40° 48’ 46” W	844 m
Psychodopygus matosi	BOLD:ACK6257	LBMI#0302	90470	AFBR423-14	KP113034	Alfredo Chaves, ES	20° 29’ 25” S	40° 57’ 28’’ W	1069 m
Psychodopygus matosi	BOLD:ACK6257	LBMI#0301	90472	AFBR422-14	KP113035	Alfredo Chaves, ES	20° 29’ 25” S	40° 57’ 28’’ W	1069 m
Psychodopygus matosi	BOLD:ACK6257	LBMI#0300	90471	AFBR421-14	KP113036	Alfredo Chaves, ES	20° 29’ 25” S	40° 57’ 28’’ W	1069 m
Psychodopygus matosi	BOLD:ACK6257	LBMI#0577	90665	AFBR740-14	KP113037	Afonso Cláudio, ES	20° 12’ 53’’ S	41° 02’ 31’’ W	1030 m
Psychodopygus matosi	BOLD:ACK6257	LBMI#0581	90669	AFBR664-14	KP113038	Afonso Cláudio, ES	20° 12’ 53’’ S	41° 02’ 31’’ W	1030 m
Psychodopygus matosi	BOLD:ACK6257	LBMI#0579	90667	AFBR662-14	KP113039	Afonso Cláudio, ES	20° 12’ 53’’ S	41° 02’ 31’’ W	1030 m
Psychodopygus matosi	BOLD:ACM6656	LBMI#0574	90662	AFBR658-14	KP113009	Afonso Cláudio, ES	20° 12’ 53’’ S	41° 02’ 31’’ W	1030 m
Sciopemyia microps	BOLD:ACK2973	LBMI#0095	90441	AFBR080-13	KP113040	Iúna, ES	20° 21’ 02” S	41° 43’ 27 W	851 m
Sciopemyia sordellii	BOLD:ACM7994	LBMI#0399	90.767	AFBR754-14	KP113043	Cáceres, MT	16° 24’ 08’’ S	57° 29’ 55’’ W	286 m
Sciopemyia sp.	BOLD:ACK6379	LBMI#0255	90414	AFBR378-14	KP113045	Pancas, ES	19° 13’ 44’’ S	40° 45’ 31’’ W	133 m
Sciopemyia sp.	BOLD:ACK6379	LBMI#0229	90371	AFBR357-14	KP113046	Pancas, ES	19° 13’ 44’’ S	40° 45’ 31’’ W	133 m
Sciopemyia sp.	BOLD:ACK6778	LBMI#0265	90351	AFBR388-14	KP112545	Pancas, ES	19° 13’ 44’’ S	40° 45’ 31’’ W	133 m
Sciopemyia sp.	BOLD:ACK6778	LBMI#0202	90370	AFBR337-14	KP113041	Pancas, ES	19° 13’ 44’’ S	40° 45’ 31’’ W	133 m

Sciopemyia sp.	BOLD:ACK6778	LBMI#0259	90413	AFBR382-14	KP113042	Pancas, ES	19° 13’ 44’’ S	40° 45’ 31’’ W	133 m
Sciopemyia sp.	BOLD:ACK6778	LBMI#0256	90415	AFBR379-14	KP113044	Pancas, ES	19° 13’ 44’’ S	40° 45’ 31’’ W	133 m
Trichophoromyia viannamartinsi	BOLD:ACM6252	LBMI#0376	90752	AFBR480-14	KP113047	Wenceslau Guimarães, BA	13° 35’ 04’’ S	39° 42’ 32’’ W	455 m
Trichophoromyia viannamartinsi	BOLD:ACM6252	LBMI#0375	90751	AFBR479-14	KP113048	Wenceslau Guimarães, BA	13° 35’ 04’’ S	39° 42’ 32’’ W	455 m
Trichophoromyia viannamartinsi	BOLD:ACM6252	LBMI#0377	90753	AFBR481-14	KP113049	Wenceslau Guimarães, BA	13° 35’ 04’’ S	39° 42’ 32’’ W	455 m
Trichophoromyia viannamartinsi	BOLD:ACM6252	LBMI#0379	90755	AFBR482-14	KP113050	Wenceslau Guimarães, BA	13° 35’ 04’’ S	39° 42’ 32’’ W	455 m
Trichophoromyia viannamartinsi	BOLD:ACM6252	LBMI#0380	90756	AFBR483-14	KP113051	Wenceslau Guimarães, BA	13° 35’ 04’’ S	39° 42’ 32’’ W	455 m
Trichophoromyia viannamartinsi	BOLD:ACM6252	LBMI#0381	90757	AFBR484-14	KP113052	Wenceslau Guimarães, BA	13° 35’ 04’’ S	39° 42’ 32’’ W	455 m
Trichophoromyia viannamartinsi	BOLD:ACM6252	LBMI#0358	90745	AFBR472-14	KP113053	Wenceslau Guimarães, BA	13° 35’ 04’’ S	39° 42’ 32’’ W	455 m
Trichophoromyia viannamartinsi	BOLD:ACM6252	LBMI#0360	90747	AFBR473-14	KP113054	Wenceslau Guimarães, BA	13° 35’ 04’’ S	39° 42’ 32’’ W	455 m
Trichophoromyia viannamartinsi	BOLD:ACM6252	LBMI#0357	90744	AFBR471-14	KP113055	Wenceslau Guimarães, BA	13° 35’ 04’’ S	39° 42’ 32’’ W	455 m
Trichophoromyia viannamartinsi	BOLD:ACM6252	LBMI#0354	90741	AFBR469-14	KP113056	Wenceslau Guimarães, BA	13° 35’ 04’’ S	39° 42’ 32’’ W	455 m
Trichophoromyia viannamartinsi	BOLD:ACM6252	LBMI#0382	90758	AFBR485-14	KP113057	Wenceslau Guimarães, BA	13° 35’ 04’’ S	39° 42’ 32’’ W	455 m
Trichophoromyia viannamartinsi	BOLD:ACM6252	LBMI#0383	90759	AFBR486-14	KP113058	Wenceslau Guimarães, BA	13° 35’ 04’’ S	39° 42’ 32’’ W	455 m
Trichophoromyia viannamartinsi	BOLD:ACM6252	LBMI#0384	90760	AFBR487-14	KP113059	Wenceslau Guimarães, BA	13° 35’ 04’’ S	39° 42’ 32’’ W	455 m
Trichophoromyia viannamartinsi	BOLD:ACM6252	LBMI#0363	90750	AFBR476-14	KP113060	Wenceslau Guimarães, BA	13° 35’ 04’’ S	39° 42’ 32’’ W	455 m
Trichophoromyia viannamartinsi	BOLD:ACM6252	LBMI#0362	90749	AFBR475-14	KP113061	Wenceslau Guimarães, BA	13° 35’ 04’’ S	39° 42’ 32’’ W	455 m
Trichophoromyia viannamartinsi	BOLD:ACM6252	LBMI#0361	90748	AFBR474-14	KP113062	Wenceslau Guimarães, BA	13° 35’ 04’’ S	39° 42’ 32’’ W	455 m
