# Supplementary material for: DNA Barcoding of Neotropical Sand Flies (Diptera, Psychodidae, Phlebotominae): Species Identification and Discovery within Brazil
Source: PLoS One. 2015 Oct 27;10(10):e0140636. doi: 10.1371/journal.pone.0140636 (PMC4624639; doi:10.1371/journal.pone.0140636)
Supplement: S3 Table — All the distances were estimated in kilometers. (BA = state of Bahia; ES = state of Espírito Santo; MG = state of Minas Gerais; MT = Mato Grosso; RJ = state of Rio de Janeiro). (PDF) [file pone.0140636.s007.pdf]

S3 Table. Suplementary data. Matrix of geographical distance between the sampling localities estimated using the Geographic Distance Matrix Generator version 1.2.3. All the distances were estimated in kilometers (BA = state of Bahia; ES = state of Espírito Santo; MG = state of Minas Gerais; MT = Mato Grosso; RJ = state of Rio de Janeiro).

	AFC	ALC	ARN	BAG	BJI	CAC	DOM	IBI	ITA	IUN	JON	LAS	MAN	MAR	PAN	SAL	SMJ	SAT	WEG
Afonso Cláudio, ES (AFC)	0.00																		
Alfredo Chaves, ES (ALC)	31.89	0.00																	
Alto Rio Novo, ES (ARN)	137.89	168.61	0.00																
Baixo Guandu, ES (BAG)	98.68	127.30	45.89	0.00															
Bom Jesus do Itabapoana, RJ (BJI)	122.02	107.3	245.54	214.83	0.00														
Cáceres, MT (CAC)	1789.24	1803.93	1771.09	1795.37	1733.95	0.00													
Domingos Martins, ES (DOM)	36.55	23.59	160.80	117.07	130.53	1822.84	0.00												
Ibitirama, ES (IBI)	75.17	77.95	182.26	155.33	65.06	1727.48	99.70	0.00											
Itaguaçu, ES (ITA)	53.76	83.88	84.76	45.35	170.18	1787.35	77.24	112.82	0.00										
Iuna, ES (IUN)	72.81	81.44	170.21	145.40	78.95	1722.51	101.48	14.33	104.40	0.00									
João Neiva, ES (JON)	72.46	89.98	106.12	60.58	193.64	1836.37	71.42	146.16	49.02	141.20	0.00								
Lagoa Santa, MG (LAS)	305.12	321.26	310.90	322.55	270.68	1484.17	339.17	247.28	306.35	240.43	355.08	0.00							
Mantenópolis, ES (MAN)	151.69	182.68	14.96	60.84	256.88	1763.83	175.42	192.97	98.98	180.48	120.97	309.01	0.00						
Marilândia, ES (MAR)	114.01	138.44	64.44	33.09	235.00	1827.46	123.02	179.32	66.54	170.87	53.92	355.65	77.62	0.00					
Pancas, ES (PAN)	113.72	141.97	38.72	15.55	230.38	1801.00	130.41	170.78	60.72	160.69	69.08	331.68	52.96	27.26	0.00				
Santa Leopoldina, ES (SAL)	55.66	60.59	139.36	93.55	167.79	1841.71	38.33	129.84	65.15	128.42	37.39	357.74	154.32	91.28	104.34	0.00			
Santa Maria de Jetibá, ES (SMJ)	35.34	58.61	113.83	70.21	157.32	1808.08	47.02	108.38	31.80	103.72	37.78	324.68	128.50	80.14	84.00	35.58	0.00		
Santa Teresa, ES (SAT)	52.78	72.01	110.22	64.62	174.23	1822.75	55.68	126.57	37.87	121.92	19.71	340.10	125.16	67.36	76.39	29.50	18.23	0.00	
Wenceslau Guimarães, BA (WEG)	751.61	780.15	616.17	653.00	860.73	1937.74	766.83	796.23	697.93	783.13	697.34	806.96	604.10	644.03	638.24	734.55	721.78	711.18	0.00
